# Co-regulation of innate and adaptive immune responses induced by ID93+GLA-SE vaccination in humans

**DOI:** 10.3389/fimmu.2024.1441944

**Published:** 2024-09-24

**Authors:** Andrew Fiore-Gartland, Himangi Srivastava, Aaron Seese, Tracey Day, Adam Penn-Nicholson, Angelique Kany Kany Luabeya, Nelita Du Plessis, Andre G. Loxton, Linda-Gail Bekker, Andreas Diacon, Gerhard Walzl, Zachary K. Sagawa, Steven G. Reed, Thomas J. Scriba, Mark Hatherill, Rhea Coler

**Affiliations:** ^1^ Vaccine and Infectious Disease Division, Fred Hutchinson Cancer Center, Seattle, WA, United States; ^2^ Infectious Diseases and Vaccines, Innovative Medicine, Johnson & Johnson, Leiden, Netherlands; ^3^ Foundation for Innovative New Diagnostics (FIND), Geneva, Switzerland; ^4^ South African Tuberculosis Vaccine Initiative (SATVI), Institute of Infectious Disease & Molecular Medicine and Department of Pathology, University of Cape Town, Cape Town, South Africa; ^5^ Department of Science and Technology/National Research Foundation (DST-NRF) Centre of Excellence for Biomedical Tuberculosis Research, South African Medical Research Council Centre for Tuberculosis Research, Biomedical Research Institute, Division of Molecular Biology and Human Genetics, Faculty of Medicine and Health Sciences, Stellenbosch University, Cape Town, South Africa; ^6^ The Desmond Tutu Human Immunodeficiency Virus (HIV) Centre, University of Cape Town, Cape Town, South Africa; ^7^ TASK, Cape Town, South Africa; ^8^ Access to Advanced Health Institute, Seattle, WA, United States; ^9^ HDT Bio Corporation, Seattle, WA, United States; ^10^ Seattle Children’s Research Institute, Center for Global Infectious Disease Research, Seattle Children’s, Seattle, WA, United States; ^11^ Department of Pediatrics, University of Washington School of Medicine, Seattle, WA, United States; ^12^ Department of Global Health, University of Washington, Seattle, WA, United States

**Keywords:** tuberculosis, vaccines, systems immunology, innate, RNA sequencing

## Abstract

**Introduction:**

Development of an effective vaccine against tuberculosis is a critical step towards reducing the global burden of disease. A therapeutic vaccine might also reduce the high rate of TB recurrence and help address the challenges of drug-resistant strains. ID93+GLA-SE is a candidate subunit vaccine that will soon be evaluated in a phase 2b efficacy trial for prevention of recurrent TB among patients undergoing TB treatment. ID93+GLA-SE vaccination was shown to elicit robust CD4+ T cell and IgG antibody responses among recently treated TB patients in the TBVPX-203 Phase 2a study (NCT02465216), but the mechanisms underlying these responses are not well understood.

**Methods:**

In this study we used specimens from TBVPX-203 participants to describe the changes in peripheral blood gene expression that occur after ID93+GLA-SE vaccination.

**Results:**

Analyses revealed several distinct modules of co-varying genes that were either up- or down-regulated after vaccination, including genes associated with innate immune pathways at 3 days post-vaccination and genes associated with lymphocyte expansion and B cell activation at 7 days post-vaccination. Notably, the regulation of these gene modules was affected by the dose schedule and by participant sex, and early innate gene signatures were correlated with the ID93-specific CD4+ T cell response.

**Discussion:**

The results provide insight into the complex interplay of the innate and adaptive arms of the immune system in developing responses to vaccination with ID93+GLA-SE and demonstrate how dosing and schedule can affect vaccine responses.

## Introduction

In 2022 (1), more people died from tuberculosis (TB) than from any other infectious disease (1), and although effective TB treatment is available and saves millions of lives, the impact on the global TB epidemic has been limited. This is, in part, because patients who successfully complete therapy with first-line drugs remain at high risk for TB recurrence (8% - 14%) due to relapse or reinfection (2, 3). Epidemiological modeling suggests that a vaccine targeting adolescents and young adults to prevent TB disease is a strategy that could achieve significant public health impact (4), making it imperative to accelerate the development of novel TB vaccine candidates. One candidate that showed promise in a recent phase 2b trial is the M72/AS01_E_ vaccine, which demonstrated 50% efficacy against TB disease in South African adults (5); the vaccine is a fusion protein of two *Mycobacterium tuberculosis* (*M.tb)* antigens co-administered with a liposome formulation of the TLR4 agonist monophosphoryl lipid A (MPL) and Quillaja saponaria Molina fraction 21 (QS21). Efforts are currently underway to more fully understand the responses elicited by the vaccine and identify the immunological correlates of protection.

The ID93+GLA-SE vaccine is also a fusion protein of four *M.tb* antigens with diverse roles in pathogenesis, co-administered with the TLR4 agonist glucopyranosyl lipid A (GLA) in a stable oil-in-water emulsion (SE) formulation (6, 7). Preclinical efficacy of therapeutic immunization with ID93+GLA-SE for prevention of recurrence has been demonstrated in mouse and non-human primate models (8, 9). A recent phase 2a study (TBVPX-203, NCT02465216) of the vaccine investigated safety and immunogenicity in 60 South African adults who had recently completed multi-drug therapy for rifampicin-susceptible pulmonary TB (10). The study evaluated three dose combinations of ID93 fusion protein and GLA-SE adjuvant, as well as two dosing schedules, with the 2 μg ID93 + 5 μg GLA-SE (“2 + 5”) dose combination given as either 2 injections with two-month spacing (injections on days 0 and 56), or 3 injections with one-month spacing (injection on days 0, 28 and 56; **Table 1**). Participants in all groups showed a robust increase in serum ID93-specific IgG. The vaccine response was also characterized by a significant increase in the ID93-specific CD4+ T cell response, with the 2-dose and 3-dose 2 + 5 regimens demonstrating comparable increases in response magnitudes after the final dose. Ultimately, the 2-dose regimen was selected for a phase 2a/2b study evaluating its efficacy in preventing TB recurrence among people living with and without HIV-1 in South Africa (NCT06205589).

**Table 1:** Study schema.

Name	Product	ID93 dose	GLA-SE dose	Dose schedule (days)			Doses	N
2 + 2 (x2)	ID93+GLA-SE	2 µg	2 µg	0	–	56	2	15
10 + 2 (x2)	ID93+GLA-SE	10 µg	2 µg	0	–	56	2	5
2 + 5 (x2)	ID93+GLA-SE	2 µg	5 µg	0	–	56	2	15
2 + 5 (x3)	ID93+GLA-SE	2 µg	5 µg	0	28	56	3	14
Placebo	Placebo	–	–	0	–	56	2	12

In this study, we applied systems vaccinology approaches to understand better the immune responses to ID93+GLA-SE. We used RNA sequencing to examine the changes in gene expression in peripheral blood that occurred after vaccination in participants in the TBVPX-203 trial. We observed evidence of both innate and early adaptive immune pathway activation within the first seven days after vaccination and showed that specific modules of vaccine-regulated genes differentiated females and males, as well as individuals in the 2-dose vs. 3-dose treatment groups. Several response modules overlapped with responses elicited by the AS01_E_ adjuvant in the M72 vaccine. Integration with previously published immune response data allowed us to interrogate further how changes in gene expression were associated with ID93-specific humoral and cellular responses.

## Methods

### Clinical trial cohort

**Figure 1:**
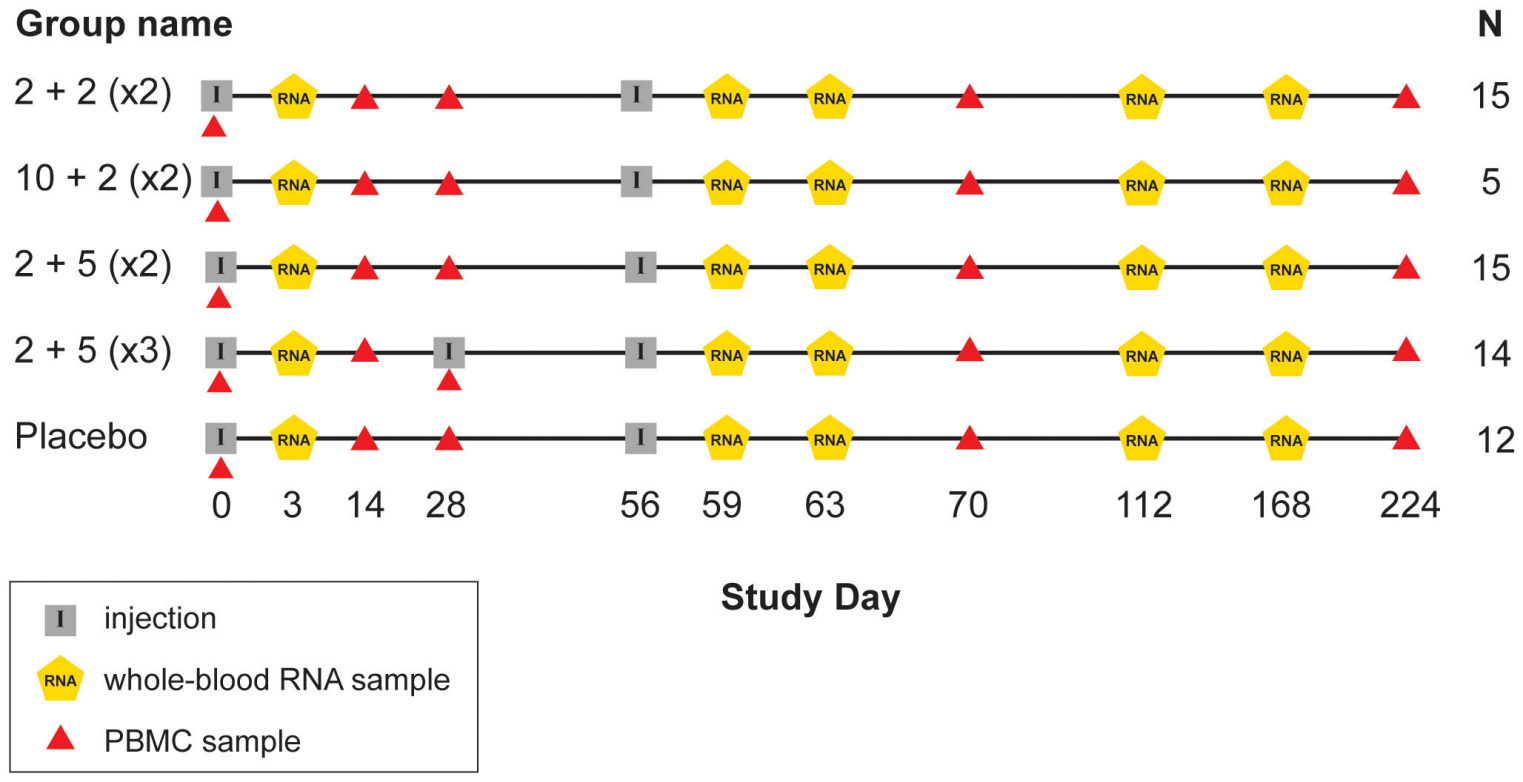
Schedule of events in TBVPX-203 study. Schematic showing the timeline of study product injections and sample collection for participants in the five study groups of TBVPX-203. The group names are defined in **Table 1** based on the dose of the ID93 antigen, the dose of the GLA-SE adjuvant and the number of injections. Schematic indicates the day of each visit relative to enrollment, and whether the visit included a product injection, a blood collection for RNA extraction or a blood collection for adaptive immune response assays.

IDRI-TBVPX-203 (ClinicalTrials.gov Identifier: NCT02465216) was a Phase 2a, randomized, double-blind, placebo-controlled, dose-escalation evaluation ID93+GLA-SE. There were two dose levels of the ID93 antigen administered in combination with one of two dose levels of GLA-SE in two different dosing regimens. The primary objective was to describe the safety and immunogenicity profile of ID93+GLA-SE in South African, HIV-negative, healthy adults who had recently completed six months of standard, four-drug (HRZE) antibiotic therapy for drug-sensitive pulmonary TB within the last 28 days. The age range of participants was 18 to 54 with a median of 27 years old. ID93+GLA-SE was given to 48 participants and 12 received placebo; one additional participant was assigned to receive vaccine, but only provided a blood sample at day 0 (**Table 1**; **Figure 1**).

### Sample processing and RNA sequencing

Ribonucleic acid (RNA) was extracted from 298 PAXgene tube samples of whole blood from 61 participants in TBVPX203. Globin mRNA was removed using bead-based hybridization (Thermofisher GLOBINclear). DNA sequencing libraries were created from polyadenylated RNA transcripts (Ilumina TruSeq, stranded). Sequencing was performed on an Illumina NovaSeq 6000 with 100 bp paired-end reads. More than 30 million reads were generated from each sample, with 70 - 85% of reads mapping to known protein coding regions [*nextflow nf-core/rnaseq* (11), STAR (12)]. Transcripts were aggregated and quantified at the gene level using *salmon* (13).

### Identification of differentially expressed genes and statistical analyses

To identify genes that were significantly increased or decreased by vaccination we used mixed-effects models of individual gene expression at pairs of time points before and after vaccination: day 3 vs. 0, day 59 vs. 56 or day 63 vs. 56. Gene counts were normalized using the trimmed mean of M-values (TMM) method (14) and log transformed. Models were fit using R package *kimma* (15) and *lme4* (16), providing “voom” precision weights (17). Linear models to detect differentially expressed genes included participant sex and accounted for the longitudinal experimental design using a random effect for within-participant covariance; as a sensitivity analysis, participant age was considered as a covariate, with only 12 genes at day 63 having an association with age (FDR-q < 0.2). Significant differential expression was based on an unadjusted-p < 0.05, FDR-adjusted q-value < 0.2 [Benjamini and Hochberg (18)] and absolute-log2 fold-change > 0.5.

Gene modules were created from the DEGs using weighted gene correlation network analysis (WGCNA) (19). A “signed correlation” distance metric was used, meaning that gene pairs with negative correlation were assigned the largest distance prior to hierarchical clustering (soft thresholding power = 7). Gene module “eigengenes” were used for subsequent analyses of treatment effects and correlations with adaptive immune responses. Descriptive plots of gene module expression used normalized counts with a mean taken across all genes in the module; in practice there was very high correlation between the module eigengene scores and normalized counts, with normalized counts being more interpretable. For statistical inference, module eigengenes were modeled using linear mixed-effects models (LMM) fit with *lme4* (16).

Gene set enrichment analysis (GSEA) was conducted using an over-representation analysis (ORA) and a hypergeometric test to understand functions associated with each of the modules. A hypergeometric test was conducted for each module using all DEGs as the “universe” of genes and testing all gene sets in the Blood Transcriptional Modules (BTMs (20); GSEA significance was based on FDR-q < 0.2. Cell deconvolution was conducted from the normalized gene counts using xCell (21). Longitudinal differences in the enrichment scores for each cell type were evaluated using a Wilcoxon signed-rank test.

Adaptive humoral and cellular immune response correlation analyses were conducted using rank-based Spearman correlation with significance based on FDRq < 0.2 to generate hypotheses. Multiplicity adjustment was computed across modules and module comparisons (e.g., days 3 vs. 0, 59 vs. 56 and 63 vs. 56). Predictive analyses were implemented using the Python package scikit-learn. Univariate model used non-penalized logistic regression while models of more than one variable used L1-penalized logistic regression. Area under the receiver operator curve was estimated using 5-fold cross-validation, with the AUC computed on the predicted outcomes for the “stacked folds”. Delong’s method (22) was used for estimating the 95% confidence interval.

### Laboratory assay data

Humoral and cellular immune response data and assay methods were previously published and described with the primary study manuscript (10). Briefly, levels of ID93-specific immunoglobulin G (IgG) in serum were measured by enzyme-linked immunosorbent assay (ELISA) using the whole fusion protein. Intracellular cytokine staining (ICS) was used to measure frequencies of ID93-specific CD4+ and CD8+ T cells from cryopreserved peripheral blood mononuclear cells (PBMCs). Antigen-specific cells were identified as those expressing at least 2 of IFNγ, IL2, TNF and/or CD154 and frequencies of these cells in the antigen stimulation condition (12-hour stimulation with whole-protein ID93) were adjusted for frequencies in the negative control condition (DMSO). There was no significant vaccine-induced increase in ID93-specific CD8+ T cells, therefore only CD4+ T cell responses were analyzed in this study. A whole-blood ICS assay (23) was also conducted with fresh whole blood and analyzed similarly to the PBMC ICS.

## Results

### Vaccination with ID93+GLA-SE induced broad changes in gene expression during the first seven days

Previously, ID93+GLA-SE was evaluated in a phase 2a trial enrolling (10) 61 volunteers who were recently diagnosed with drug-susceptible pulmonary tuberculosis and who successfully completed six months of standard TB therapy. Whole-blood samples were provided by participants at visits scheduled on the day of the first injection (day 0) and at subsequent visits including study days 3, 56, 59, 63, 112 and 168. To study the transcriptional profile of samples before and after vaccination, blood was processed and submitted for RNA sequencing, with resulting transcript sequences mapped to the human genome and quantitated at the gene level for further analysis (see Methods).

**Figure 2:**
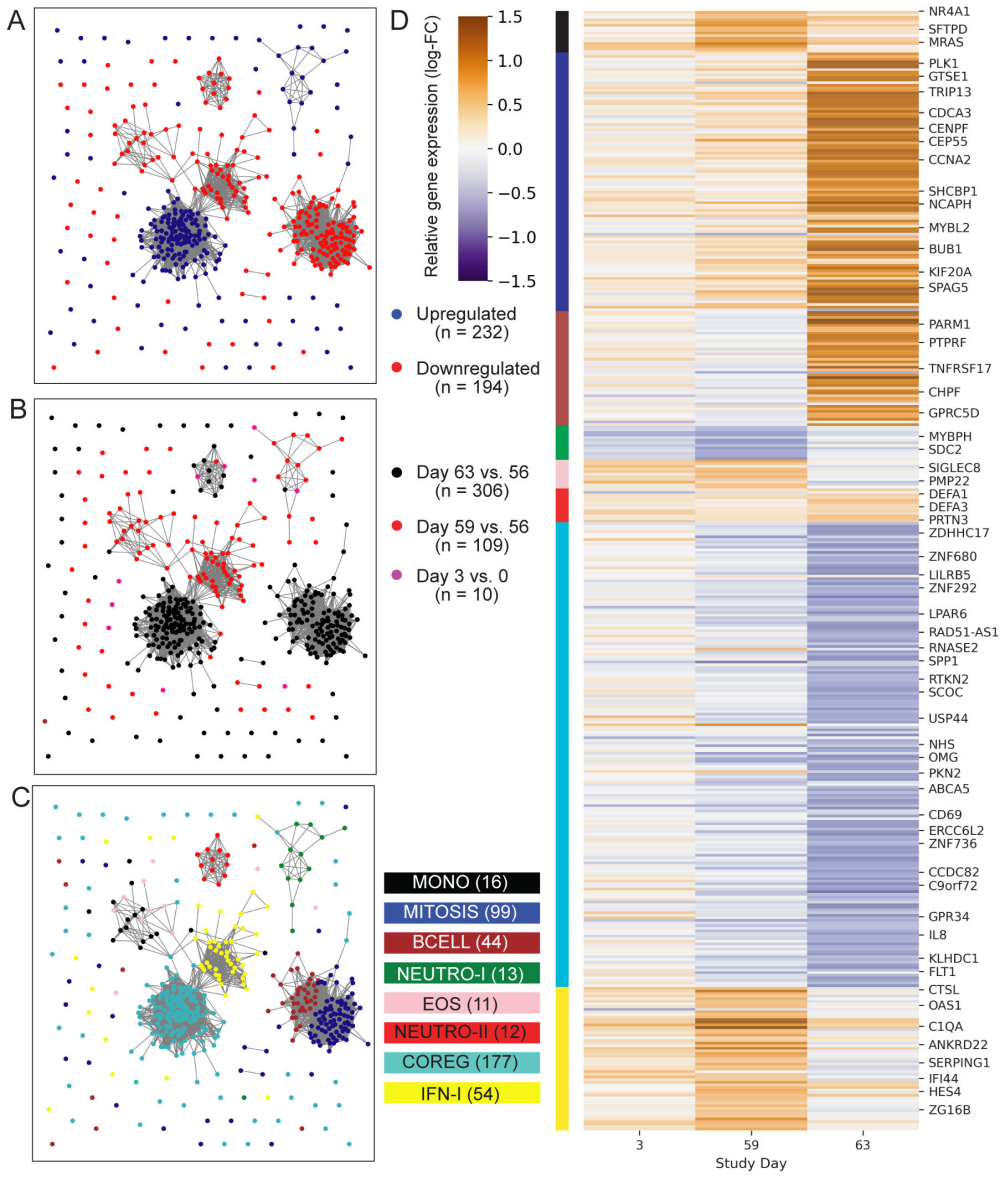
Profile of ID93+GLA-SE differentially expressed genes. Network plots represent 426 genes (nodes) that were differentially expressed after ID93+GLA-SE vaccination. Significant differential expression was based on an unadjusted-p < 0.05, FDR-adjusted q-value < 0.2 and absolute-log2 fold-change > 0.5. Edges connecting nodes represent a positive rank correlation with R > 0.6 (edge length does not convey additional information). Node color indicates **(A)** up- (red) vs. down- (blue) regulation relative to pre-vaccination, **(B)** differential regulation at one of three time points: days 3 vs. 0 (pink), 59 vs. 56 (red) or 63 vs. 56 (black), or **(C)** membership in one of eight gene modules created using WGCNA. Genes that were differentially expressed at more than one time point are colored for the time point with the largest fold-change, therefore there are fewer genes plotted than total DEGs stated in results. Each gene is in exactly one module. **(D)** Heatmap indicates log-transformed fold-change for each differentially expressed gene, relative to the most recent vaccine dose (either day 0 or day 56). Module membership is indicated by the color bar. A subset of genes are labeled by gene symbol including some of those cited in the text or found in gene sets that were significantly enriched with DEGs in the GSEA analysis.

Initial analysis to identify vaccine-induced gene expression was focused on the two treatment groups that received the 2 ug ID93 + 5 ug GLA-SE dose: one group received two injections on days 0 and 56 while the other received three injections on days 0, 28 and 56. To identify genes that were differentially expressed following an injection, we modeled three pre- versus post-vaccine comparisons: days 3 vs. 0, 59 vs. 56 and 63 vs. 56. Pooling individuals from the two treatment groups, we identified differentially expressed genes (DEGs) using a linear mixed-effects model that accounted for covariation in paired samples from the same individual and adjusted for participant sex at-birth. In total the models identified 241 upregulated and 208 downregulated genes based on the pre-specified significance criteria (**Figure 2**; unadjusted-p < 0.05, FDR-q < 0.2, |log2-fold change| > 0.5) for a total of 426 unique DEGs. Despite receiving an identical vaccination three days earlier, participants had fewer genes with a significant change from day 0 to day 3 (12 DEGs) compared to those with a significant change from day 56 to day 59 (122 DEGs; **Supplementary Figure S1**); notably, nearly all of the genes that were upregulated at day 59 were also upregulated at day 3, albeit with a smaller fold-change (**Supplementary Figure S1**). Substantially more genes were found differentially expressed at day 63 vs. 56 with 168 genes downregulated and 145 genes upregulated (**Supplementary Data Sheet 1**).

To better understand the functional signaling patterns among the differentially expressed genes we used weighted gene correlation network analysis [WGCNA (19)], which identified eight distinct modules of coregulated genes that aided interpretation and enabled downstream analysis (**Table 2**; **Supplementary Data Sheet 1**). These patterns of correlated gene expression were also easily visible from rank correlation networks formed by connecting genes with a correlation coefficient, R > 0.6 (**Figure 2**).

**Table 2:** Summary of gene modules associated with ID93+GLA-SE vaccination.

Module	N	Day 3	Day 59 (+3*)	Day 63 (+7*)	Possible role
MONO	12	UP	UP	UP	monocyte genes, surfactant gene
EOS	11	UP	UP	–	eosinophil genes
IFN-I	54	UP	UP	–	antiviral, type I IFN, complement receptors and dendritic cells
NEUTRO-I	13	DOWN	DOWN	–	neutrophil genes
MITOSIS	99	–	–	UP	cell cycle, transcription
BCELL	44	–	–	UP	B cells, plasma cells and Ig genes
NEUTRO-II	12	–	–	UP	neutrophil granules, defensins
COREG	177	–	–	DOWN	genes coregulated other modules

* +3 and +7 refer to the number of days post vaccination.

### Cell-specific innate immune genes were differentially regulated three days after vaccination

**Figure 3:**
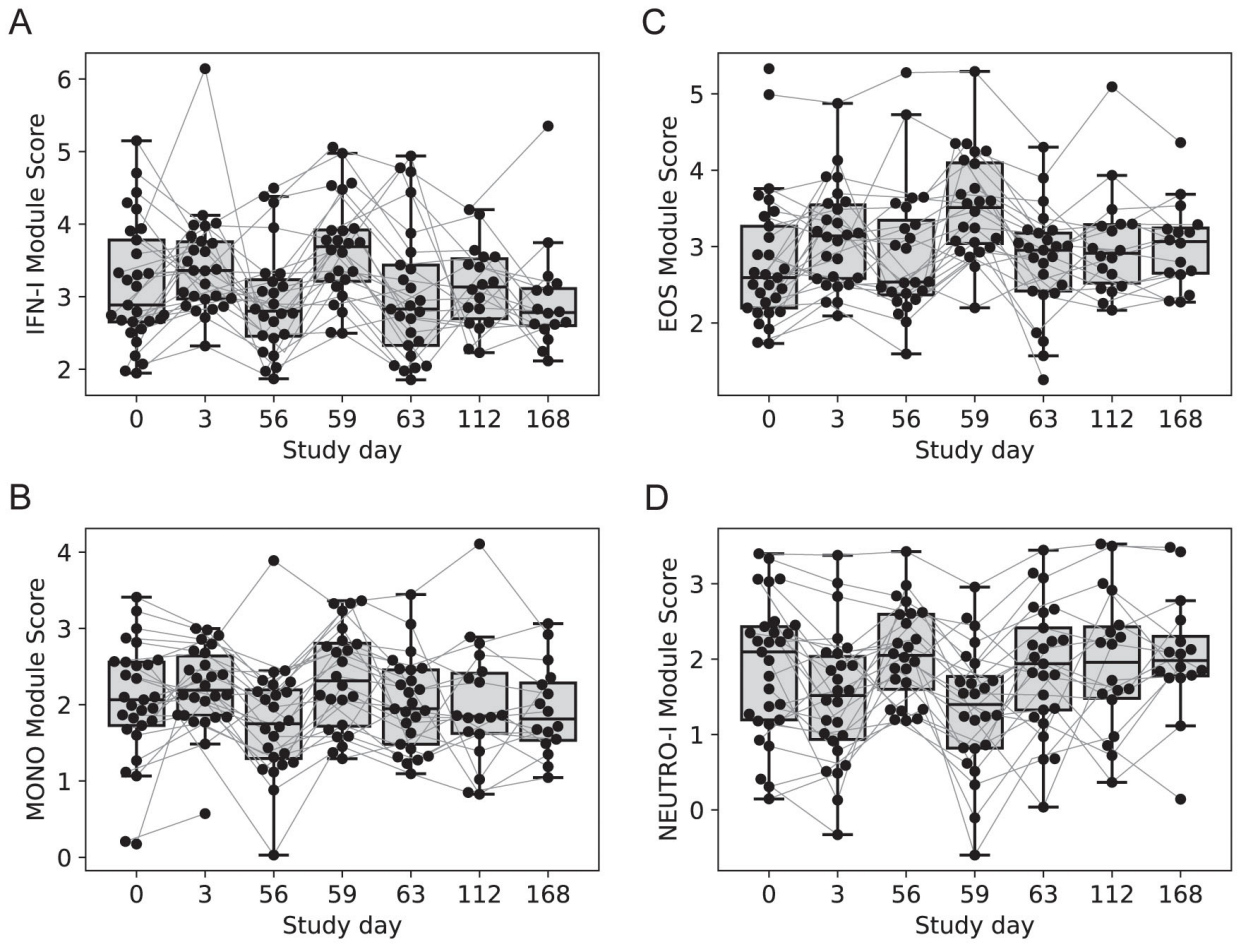
Longitudinal expression of modules with changes 3-days post-vaccination. Boxplots of the eigengene expression score for each participant in either the 2-dose or 3-dose “2 + 5” ID93+GLA-SE vaccine group (n=29). **(A)** IFN-I module, **(B)** MONOS module, **(C)** EOS module, **(D)** NEUTRO-I module. A line for each participant is overlaid, connecting longitudinal observations. Box extents indicate the interquartile range (IQR) with whiskers indicating the extent of the most extreme observation within1.5-times the IQR.

Four modules contained genes typically associated with innate cell types that were differentially regulated at day 3 vs. 0, day 59 vs. 56 or both; average expression for the latter returned to pre-vaccine levels by day 63. One module contained 54 genes including several associated with innate antiviral responses and the type I interferon pathway, with OAS1, BATF2, GPB5 and SERPING1 having high centrality scores within the module network (referred to as IFN-I module, **Figure 3A**) (23, 24). A gene set enrichment analysis (GSEA) of genes from the IFN-I module using gene sets from the blood transcriptional modules [BTMs (20)] also detected enrichment for sets involved in complement activation (M112.0), complement and other receptors in DCs (M40), immune activation (M37.0), and activated dendritic cells (M67), in addition to the antiviral IFN signature (M75) (all with FDR-q < 0.2).

A second module contained 12 genes, several of which were found previously to be associated with subsets of monocytes including MRAS, CDKN1C, LYPD2, LYNX1, VMO1 and SFTPD (referred to as MONO module). Notably, surfactant protein D (SFTPD) is typically expressed by alveolar macrophages in the lung and may be involved in lung defense. The MONO module was significantly upregulated at day 59 vs. day 56 (**Figure 3B**). A third module contained 11 coregulated genes that were upregulated 3 days after vaccination; all 11 genes were most highly expressed by eosinophils (EOS module, **Figure 3C**) [Human Protein Atlas (24)]. The fourth module contained 13 genes that were downregulated three days after vaccination and were almost all primarily expressed by neutrophils (HPA), including the most highly expressed genes AOC3, OTX1, SDC2 and TP35INP2 (NEUTRO-I module, **Figure 3D**).

With any changes in gene expression observed from whole tissues, they could be linked to a change in the cellular composition of the tissue and/or a change in regulation within cells. To address these hypotheses, we used computational cellular deconvolution, which leverages a database of cell-type specific gene expression patterns to infer enrichment scores that are proportional to the relative abundance of cells composing the sample [xCell (21)]. The output of xCell is an enrichment score that can be used for a longitudinal comparison for a given cell type, but cannot be used to infer the absolute frequency of cell types in a sample, and therefore cannot be used to compare across cell types. Generally, scores were highly variable and changes were small (**Supplementary Data Sheet 1**), however the analysis suggested that there was a 2.5-fold increase in the score for dendritic cells from days 56 to 59 (p=0.0012, FDRq=0.038); there was a similar trend for eosinophils and monocytes that was not significant (**Supplementary Figure S2**). The relative score for neutrophils in the blood did not substantially change across days 56 – 63 (**Supplementary Figure S2**), supporting the hypothesis that the decrease in expression of NEUTRO-I genes was related to downregulation of a subset of genes expressed by neutrophils. The analysis was repeated with an additional deconvolution method CIBERSORT (**Supplementary Figure S3**), which did not identify any cell subsets that were significantly changed by vaccination (**Supplementary Data Sheet 1**); this is consistent with the hypothesis that genes in transcript abundance could be attributed to changes in gene expression as opposed to changes in the abundance of specific cell types, but it remains possible that the variability associated with estimating changes in cellular composition made it difficult to detect significant changes.

### ID93+GLA-SE transiently increased expression of genes associated with TB disease progression

Many studies have demonstrated that expression levels of specific transcripts in whole-blood can be used to predict TB disease progression as well as treatment response (25–29). Given the vaccine-induced changes in the IFN-I module genes – genes that are also included in TB risk signatures – we looked at the kinetics of two leading risk signatures across study participants, through 6 months post-vaccination (**Supplementary Figure S4**). Both the Sweeney3 signature (GBP5, DUSP3, and KLF2) and the Darboe11 (a.k.a., RISK11: BATF2, ETV7, FCGR1C, GBP1, GBP2, GBP5, SCARF1, SERPING1, STAT1, TAP1, and TRAFD1) signatures were transiently increased 3 days after the first dose and booster dose. Expression levels returned to baseline by day 7 post-boost (i.e., day 63) and remained at consistent levels through day 168.

### Genes associated with T and B cell responses were upregulated three and seven days after vaccination

**Figure 4:**
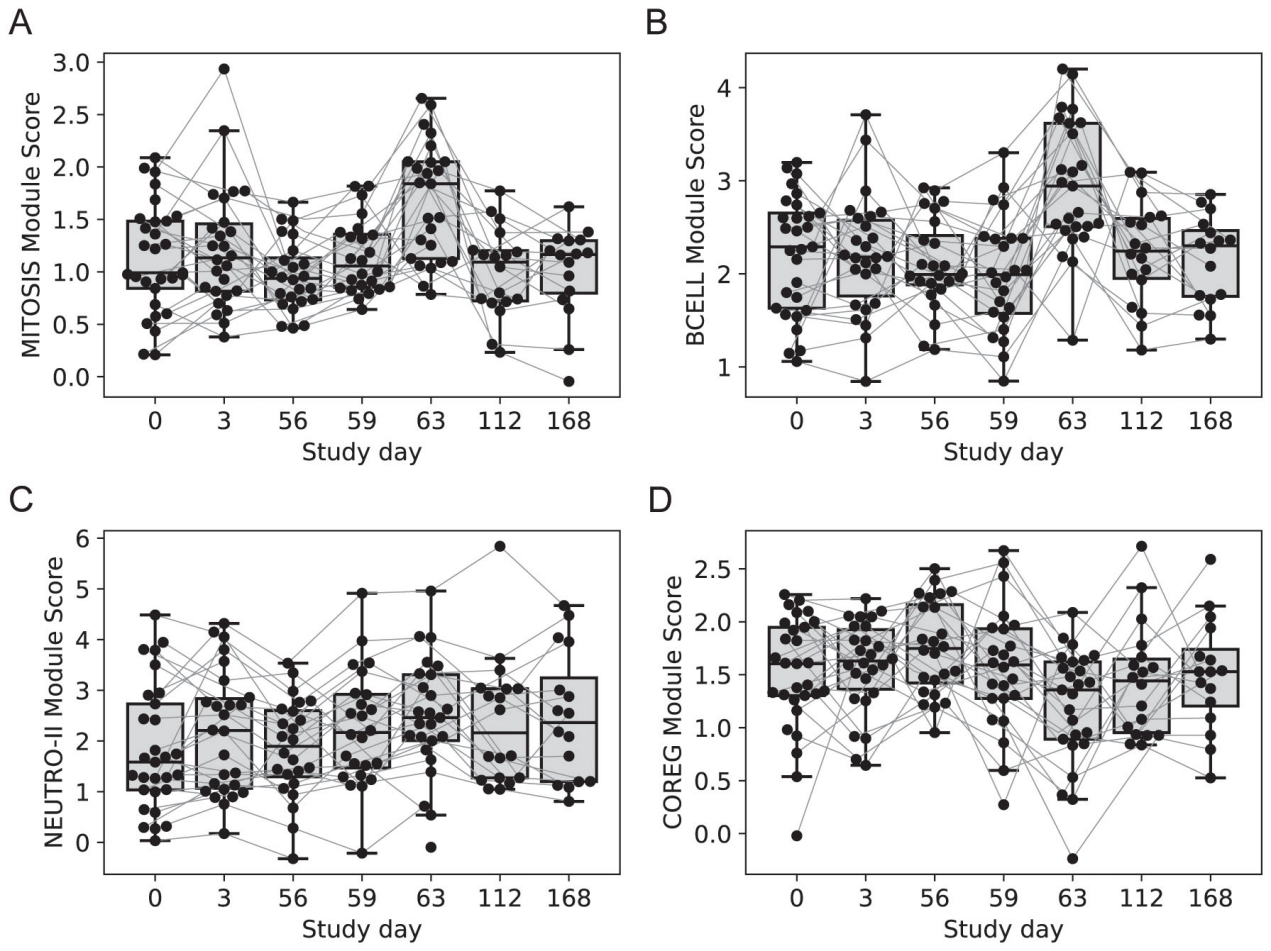
Longitudinal expression of modules with changes 7-days post-vaccination. Boxplots of the eigengene expression score for each participant in either the 2-dose or 3-dose “2 + 5” ID93+GLA-SE vaccine group (n=29). **(A)** MITOSIS module, **(B)** BCELL module, **(C)** NEUTRO-II module, **(D)** COREG module. A line for each participant is overlaid, connecting longitudinal observations. Box extents indicate the interquartile range (IQR) with whiskers indicating the extent of the most extreme observation within1.5-times the IQR.

Two gene modules had similar average expression profiles showing a significant increase at day 63 vs. day 56, seven days after the last injection (**Figures 4A, B**; no samples were available seven days after the first injection). However, the genes formed two distinct modules based on their covariation, with a “MITOSIS” module consisting of 99 genes enriched in gene sets associated with mismatch repair, cell cycle and transcription, and a “BCELL” module containing 44 genes enriched in gene sets associated with B cells, plasma cell surface markers and immunoglobulin genes (**Supplementary Data Sheet 1**). The cell composition deconvolution analysis suggested that the relative frequency of class-switched memory B cells (p=0.0040, FDR-q=0.063; **Supplementary Figure S2**) and type-1 helper T cells (Th1, p=0.0012, FDRq=0.038), were increased at day 63 vs. day 56. A third smaller module of 12 tightly coordinated genes was also significantly increased at day 63 and contained genes associated with neutrophil cytotoxic granules (NEUTRO-II module; **Figure 4C**), including three genes encoding defensins (DEFA1, DEFA3 and DEFA4). The module also included lactotransferrin (LTF) and PRTN3, which are also expressed primarily by neutrophils.

### Genes downregulated after vaccination were negatively correlated with upregulated modules

**Figure 5:**
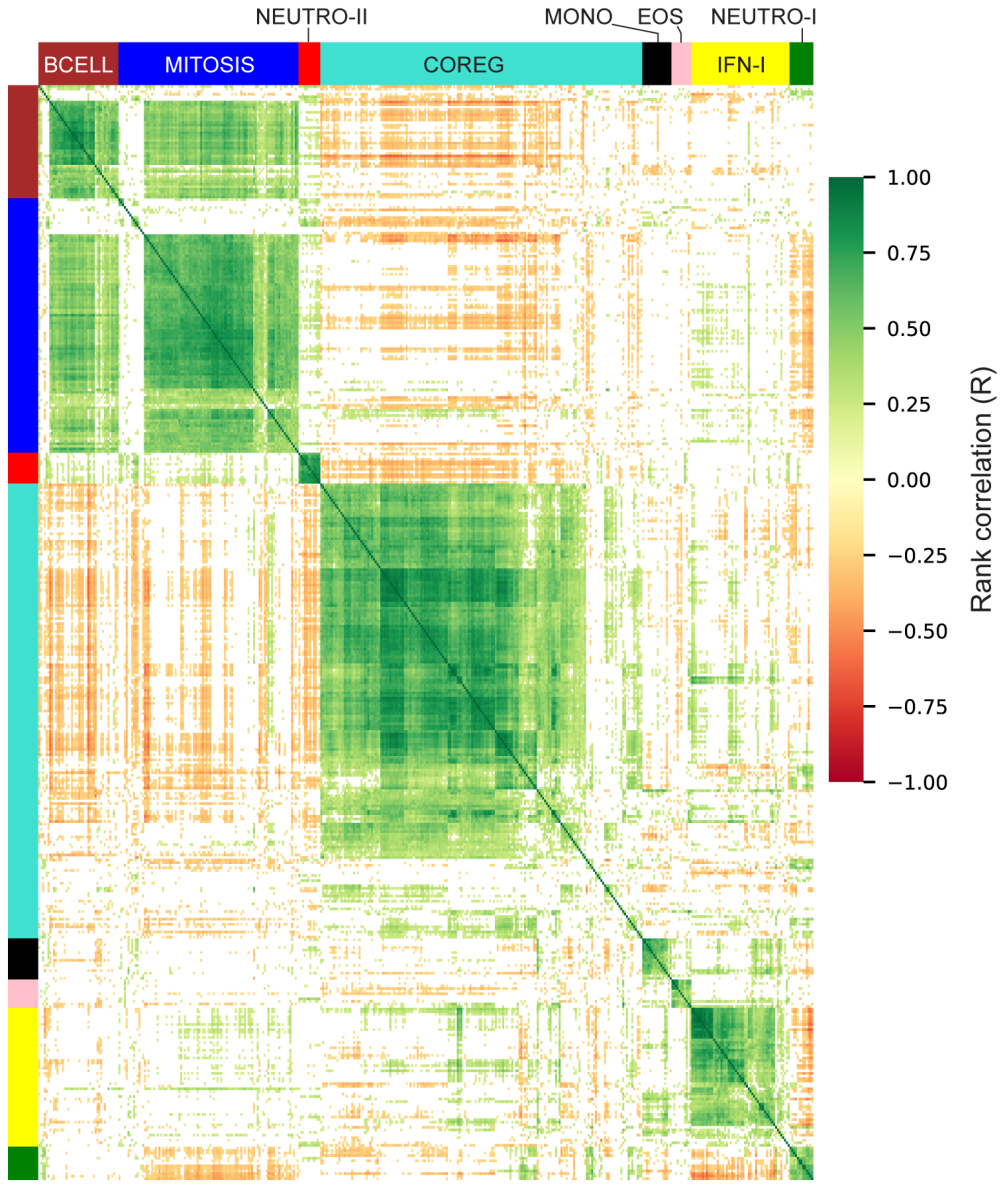
Correlation heatmap of differentially expressed genes. Rank correlations were estimated for all pairs of differentially expressed genes. Genes are organized by module and hierarchically clustered within each module. Color bars indicate module membership. Correlations with abs-R < 0.2 are censored for clarity (white).

The gene modules constructed using WGCNA have low correlation across individuals by design; genes with positively correlated expression levels were grouped into the same modules leaving relatively little correlation between modules. However, genes that were inversely/negatively correlated had large distances in the network (and module construction steps) and therefore formed distinct modules. As a result, we have two distinct modules that decreased with vaccination: NEUTRO-I which decreased 3 days after vaccination and COREG, a large module of 177 genes that decreased at day 63 (7 days post-boost; **Figure 4D**). While the COREG module contained several immune-related genes including (e.g., LILRB5, IL8, CD69), GSEA did not identify any significantly enriched gene sets, nor could we identify a single function that could relate all the genes. Instead, it appears that the COREG module contains genes that are coregulated with genes in other modules that were increased at day 63 (**Figure 5**). For example, 70 COREG genes (40%) have a negative rank correlation coefficient, R< -0.5 with at least 1 gene from the BCELL module; similarly, 33 COREG genes are negatively correlated with at least one MITOSIS gene. In comparison, only 3 genes are negatively correlated with any of the MONO, EOS or IFN-I genes, which are increased at day 3.

### IFN-I and EOS genes responded more robustly in female participants

With genes grouped into modules, we sought to understand how early vaccine-induced gene expression changes were associated with the different vaccine regimens, participant sex and ultimately, the humoral and cellular responses that were evaluated weeks later. To do this, we used the eigengene of each module as a longitudinal measure of expression for each participant and evaluated associations with additional variables using linear mixed-effects models (LMM). As an initial check, we analyzed participants in the “2 + 2 (x2)” treatment group which received lower doses of GLA-SE (2 μg, n=15). We noted similarities with the groups that received 5 μg of GLA-SE, that had been used to identify DEGs, though responses were lower (**Supplementary Figure S5**). For example, among participants in the “2 + 2 (x2)” group the IFN-I module was increased at days 3 and 59, while the BCELL and MITOSIS modules were increased at day 63; these were consistent with the changes seen in the 5 μg GLA-SE dose groups; these findings served as a partial validation of our approach to identify genes that were induced by vaccination.

**Figure 6:**
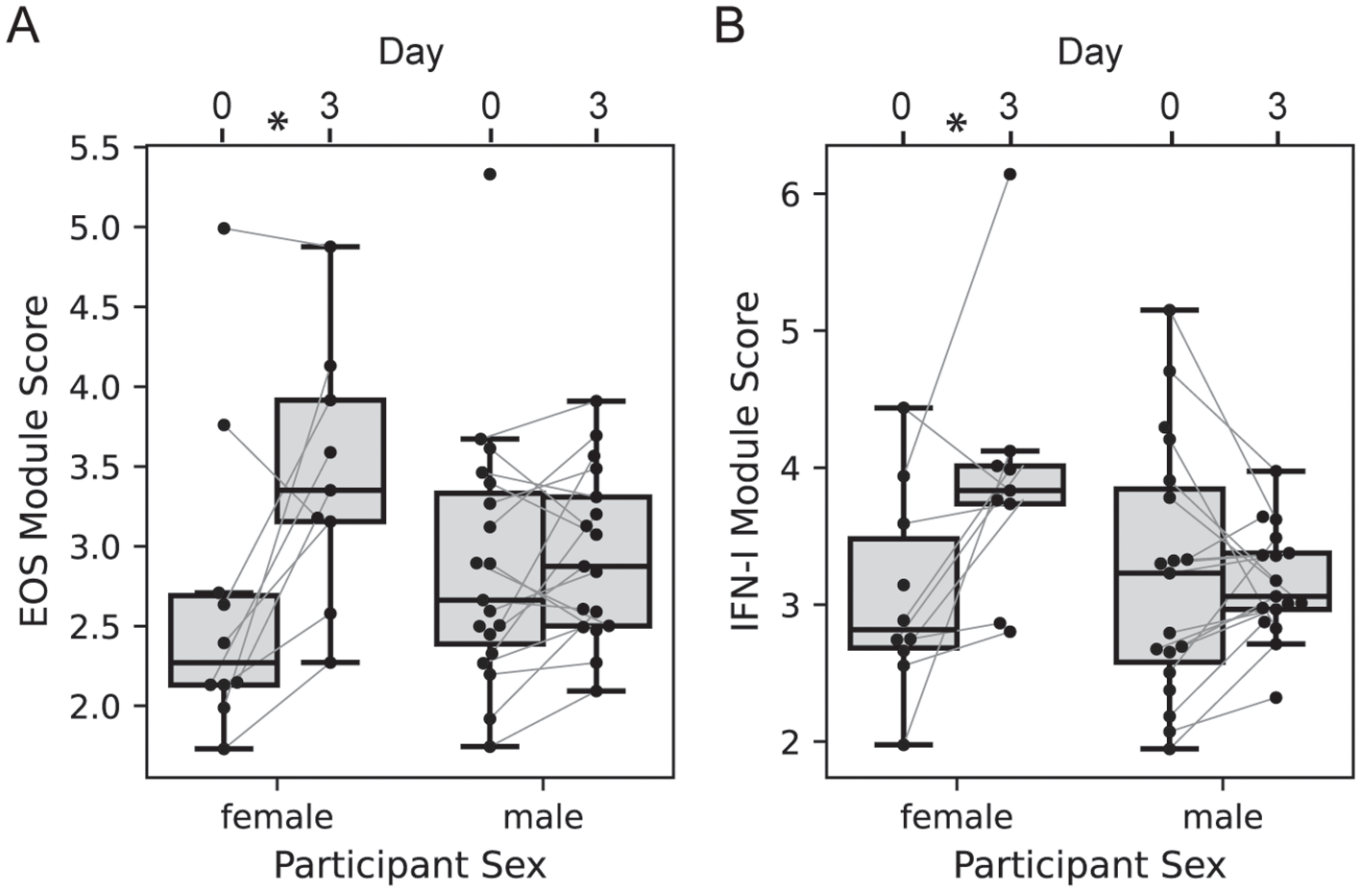
Participant sex modifies day 3 transcriptional response. Boxplots of the EOS **(A)** and IFN-I **(B)** module expression scores for each participant in either the 2-dose or 3-dose “2 + 5” ID93+GLA-SE vaccine group (n=29). Module expression score is the log-normalized transcript counts with a mean taken across all genes contained in the module. Scores are organized by visit with lines connecting the scores of an individual participant at day 0 and day 3, and by sex (n=10 of 29 female). Linear mixed modeling showed a significant modification of the vaccine-induced change by sex (EOS p = 0.022; IFN-I p=0.012).

We next evaluated the effect of participant sex on vaccine transcriptional responses; the 2-dose and 3-dose “2 + 5” groups were pooled with responses analyzed at day 3 vs. 0, since the treatment groups received identical treatments at day 0 (n=10 of 29 female). The responses of EOS and IFN-I genes at day 3 were significantly higher among females versus males (**Figure 6**, LMM p = 0.022 and 0.012, respectively). There was no significant effect of sex on the MONO or NETRO-I modules and we did not test the modules that were unchanged at day 3.

Testing for an effect of sex on vaccine responses was more difficult at days 59 and 63, since the treatment groups differed and were not well-balanced by sex; the 2-dose group had 8 of 15 female participants, while the 3-dose group had 2 of 14 female participants, leading us to focus on the 2-dose group. At 3 days post-2^nd^ vaccination we saw an increase in EOS and IFN-I genes that tended higher among females, but was not significantly different (LMM p = 0.109 and 0.318, respectively). There was no effect of sex on the other modules at the day 59 and day 63 visits.

### Intervening day 28 vaccination blunted immune response to the booster vaccination

In the primary study, the 2-dose and 3-dose vaccine regimens had distinct cellular and humoral responses. The difference was most salient at day 70: 14 days after the 2^nd^ dose for one group and 14 days after the 3^rd^ dose for the other. Though the absolute levels of ID93-specific IgG and CD4+ T cells were comparable at day 70, there was a large increase from day 56 to day 70 in the 2-dose group that was not seen in the 3-dose group (reproduction of data in **Supplementary Figures S6, S7**). We hypothesized that there would be a similar reduction in the gene expression responses of the 3-dose group. We modeled the expression levels of each module at days 56, 59 and 63, including treatment group and sex as potential modifiers of the response; the interaction term between treatment group and day was the indicator of whether there was a difference in the 2-dose vs. 3-dose response.

**Figure 7:**
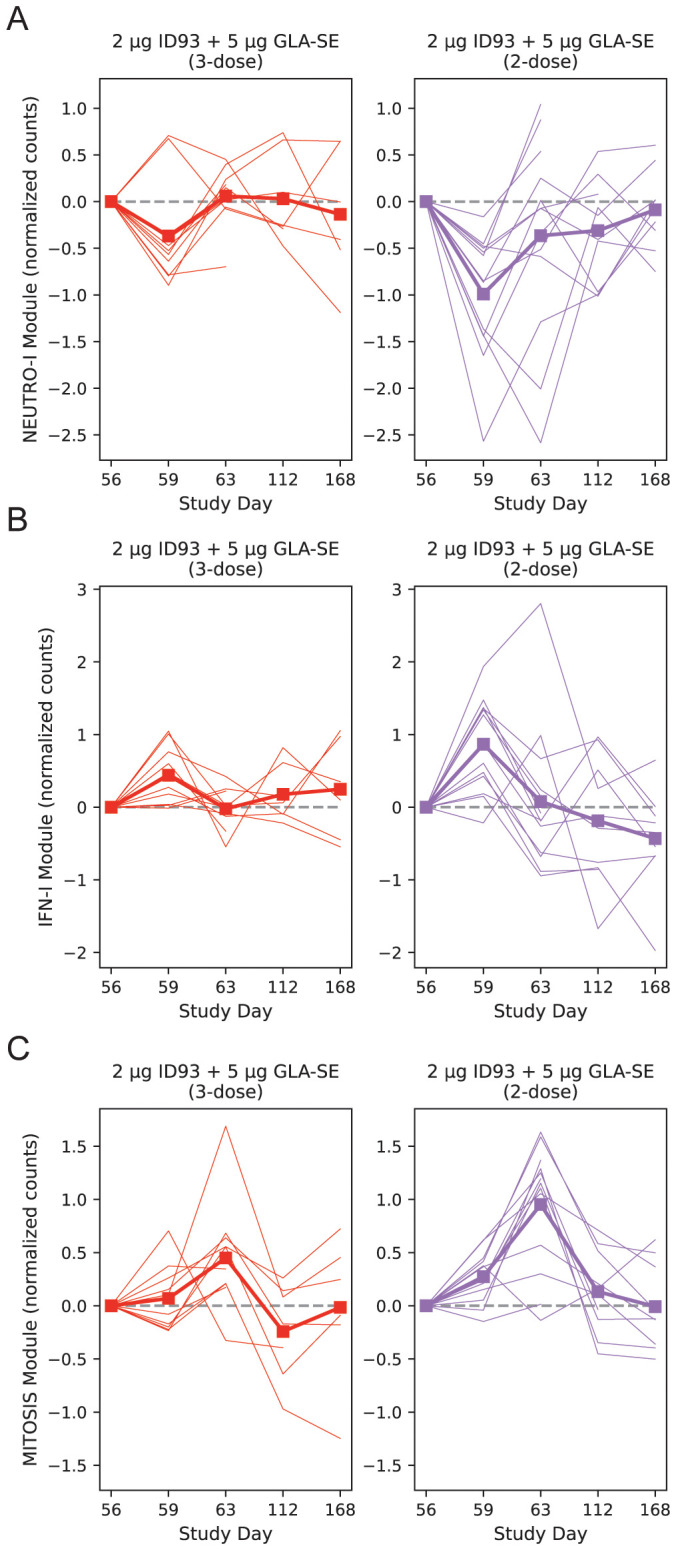
Post-boost transcriptional changes by treatment group. Baseline-subtracted normalized expression level plotted for the NEUTRO-I **(A)**, IFN-I **(B)** and MITOSIS **(C)** modules, organized by participant and day. A module’s expression is the log-normalized transcript counts with a mean taken across all genes contained in the module; each participant’s module expression at day 0 is subtracted from the time series for that individual. Module expression values are plotted longitudinally with each participant represented by a single line and the group mean represented by a thick line and square symbols (treatment group indicated at the top of each panel). Linear mixed modeling showed that the 2-dose group had a greater change at day 59 vs. 56 for the NEUTRO-I module (p=0.049) with a trend for the IFN-I module (p=0.094). At day 63 vs. day 56 the MITOSIS module had a larger response in the 2-dose group (p=0.0013). Dashed line on each plot indicates zero change from day 0 (i.e., pre-vaccine) expression. Broken lines represent missing data that result from missing samples.

With the model of day 59 vs. 56 we noted that the NEUTRO-I module differed significantly in the two treatment groups after adjustment for participant sex, having a 2.6-fold greater decrease in the 2-dose vs. 3-dose group (**Figure 7A**, p = 0.049). Similarly, the IFN-I module tended towards a greater response in the 2-dose group (**Figure 7B**, p = 0.094). The changes in the other modules were similar across the groups. At day 63 the MITOSIS module was increased in both groups, but the response was 2.25-fold greater in the 2-dose group (**Figure 7C**, p=0.0013). The BCELL group showed a similar trend, but it was not significant (p=0.115). To assess if the blunting of the vaccine response in the 3-dose group could be explained by a difference in gene expression of the modules at the time of the last vaccination, we tested for an association between module score at day 56 and the treatment group, adjusting for sex; none of the modules were significantly different across the groups at day 56 (**Supplementary Data Sheet 1**).

### Early innate transcriptomic signature correlated with ID93-specific CD4+ T cell responses

To better understand the orchestration of innate and adaptive immune responses after vaccination we conducted a correlation analysis to identify the gene expression modules that were associated with the magnitude of the antigen-specific CD4+ T cell and IgG antibody responses (**Supplementary Data Sheet 2**). The ID93-specific CD4+ T cell response was measured using two intracellular cytokine staining (ICS) assays that computed the proportion of cells expressing cytokine after ID93 stimulation: (1) PBMC-ICS using cryopreserved peripheral blood mononuclear cells (PBMC, **Supplementary Figure S6**) and (2) WB-ICS using fresh whole-blood. The level of ID93-specific IgG was measured by ELISA and reported as mean endpoint titers (MEPT, **Supplementary Figure S7**).

With the hypothesis that changes in post-vaccine gene expression were correlated with the adaptive responses we focused on the gene module changes at specific time points that were significantly increased or decreased (**Table 2**, 13 significant comparisons). Correlations were computed between the change in gene expression versus the absolute CD4+ T cell or IgG response level at days 14, 28, 70 or 224.

**Figure 8:**
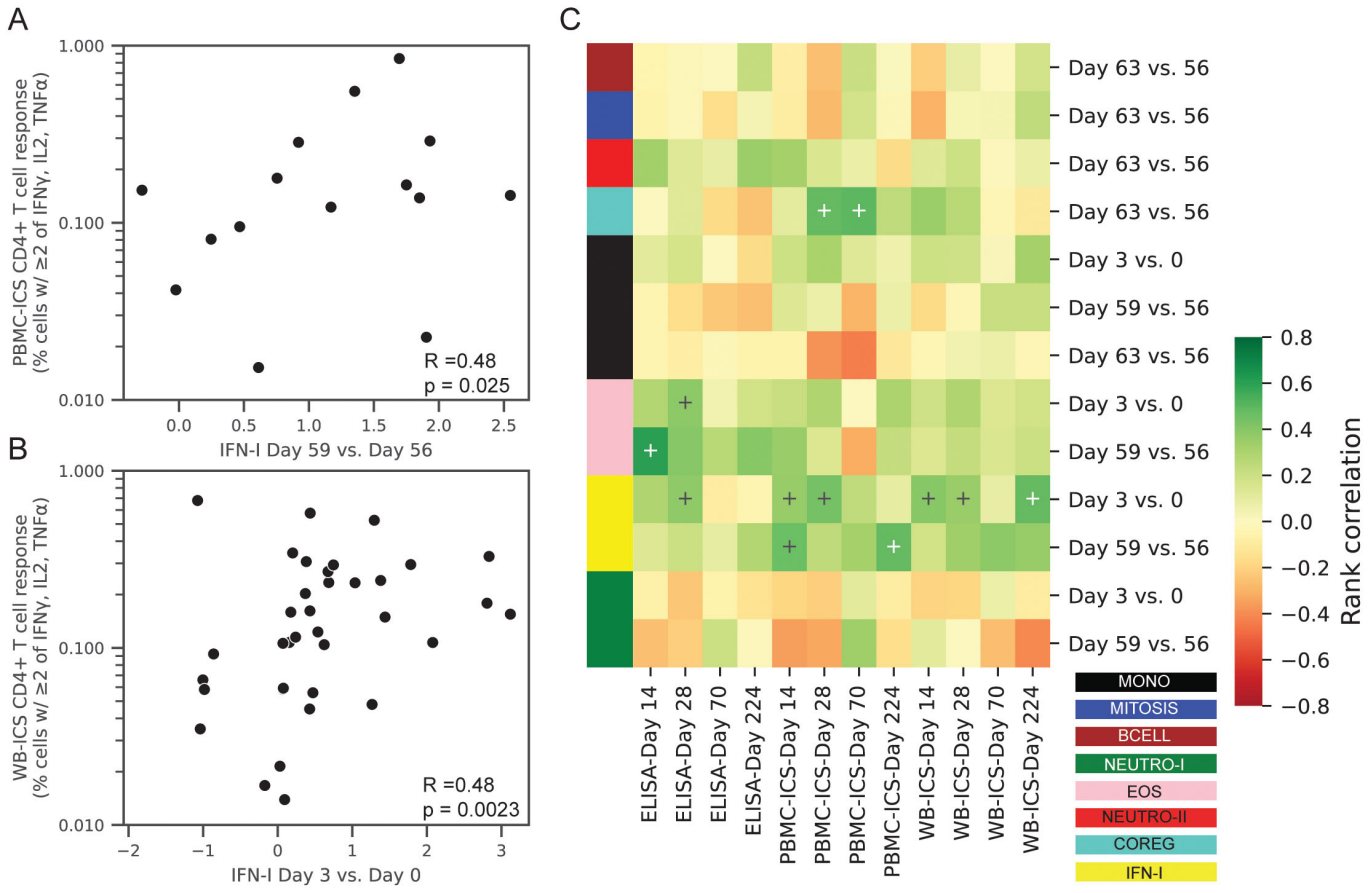
Correlations between transcriptional responses and ID93-specific adaptive responses. Rank correlations between pairs of gene module expression changes and absolute levels of ID93-specific CD4+ T-cell responses and IgG antibody levels in the blood. Transcriptional changes were expressed as changes in the eigengene score between two days (e.g., day 3 vs. 0). CD4+ T-cell responses were measured by PBMC-ICS or WB-ICS while IgG was measured by ID93 ELISA. Significant correlations (FDRq < 0.2 and p<0.05) included **(A)** PBMC-ICS at day 224 with IFN-I at day 59 vs. 56 and **(B)** WB-ICS at day 224 and IFN-I at day 3 vs. 0 (rank correlation coefficient R and unadjusted p-valued indicated in lower left corner of panel). **(C)** Heatmap of all tested correlations organized by module color (see **Figure 1** for key). Modules were only tested for correlation at the day that they showed a significant change post-vaccination (13 comparisons). Crosses on heatmap indicate an unadjusted p < 0.05 (black and white for visual clarity).

After adjusting for multiple comparisons, we found that the change in the IFN-I module at day 3 was significantly associated with ID93-specific CD4+ T-cell response at day 14 (WB-ICS, rho=0.39, FDRq=0.11), day 28 (PBMC-ICS, rho=0.44, FDRq=0.19) and day 224 (WB-ICS, rho=0.48, FDRq=0.058). Looking more broadly, these correlations were representative of a generally positive correlation between the IFN-I responses at day 3 and the CD4+ T-cell response (**Figure 8**). There was also a positive correlation between the day-3 IFN-I (rho=0.38) and EOS (rho=0.37) modules with the ID93-specific IgG level at day 28, though it did not reach the significance criteria (FDRq=0.212, FDRq=0.212 respectively).

Directly comparing ID93-specific CD4+ T cell and IgG responses we found that female participants had higher PBMC-ICS CD4+ T cell responses at day 28 (unadjusted-p = 0.032) and higher ELISA IgG MEPT at day 14 (unadjusted-p = 0.039), however no significant differences were observed at any other visits (**Supplementary Figure S8**). Computing partial correlations adjusted for participant sex reduced the correlations with the IgG response (e.g., IFN-I partial-rho=0.28), but did not consistently reduce the correlations with the CD4+ T cell response (**Supplementary Figure S9**). This suggests that the correlations were not primarily driven by the effect of sex on both innate and adaptive responses.

### Post-boost transcriptomic signature correlated with subsequent boosting of antibody and T-cell responses

We repeated the correlation analysis focused on responses after the boost (day 56), replacing absolute levels of CD4+ T-cell responses and IgG with relative responses, subtracting the response observed prior to the boost. While none of the correlations had FDRq < 0.2, the BCELL and MITOSIS modules at day 63 had low to moderate positive correlations with all the adaptive immune responses at days 70 and 224 (**Supplementary Figure S9**). This is consistent with the 3-dose vs. 2-dose treatment effect, with the 3-dose group having lower BCELL and MITOSIS responses and little or no increase in antibody and T cell responses with the 3^rd^ dose. Notably, the IFN-I module was no longer associated with these adaptive responses measured relative to day 56.

A similar sensitivity analysis was conducted substituting absolute levels of CD4+ T cell and IgG at days 14 and 28 with responses relative to pre-vaccination (day 0) levels to assess if correlations were more strongly associated with relative changes in these responses (**Supplementary Figure S9**). All of the associations between IFN-I and the adaptive responses were weaker with the baseline-subtracted responses.

### IFN-I module responses predictive of high CD4+ T-cell responders at day 224

**Figure 9:**
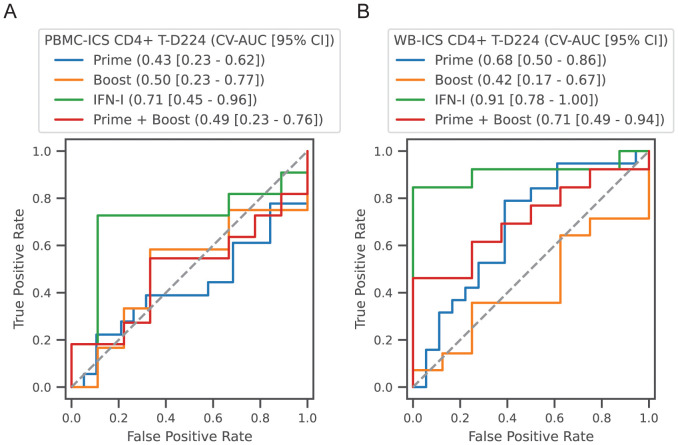
Classification of high versus low CD4+ T-cell responders. Participants in all vaccine groups were categorized as high or low based on the median of the CD4+ T-cell response; **(A)** PBMC-ICS and **(B)** WB-ICS responses were analyzed separately. Regularized logistic regression (L1 penalty) was used to train a classifier based on feature sets: (1) Prime: modules that responded after the 1^st^ dose (4 features), (2) Boost: modules that responded after boost dose (i.e., day 56, 9 features), (3) IFN-I modules after the prime and boost (2 features), (4) Combination of all prime and boost modules (13 features). Classifier performance was evaluated in 5-fold cross-validation (CV). Receiver operator curves (ROC) are plotted for each feature set, with cross-validated area under the ROC curve (CV-AUC) and 95% confidence interval (CI) indicated above the plot. Gray dashed line indicates performance equivalent to uninformed guessing.

To complement the correlation analysis we also conducted a predictive analysis, employing multivariate regularized logistic regression modules to predict high and low responders. High versus low response was defined as above or below the median response. Predictor sets included, (1) Prime: all modules with a day 3 response (see **Table 2**, 4 variables), (2) Boost: all modules with a day 59 or day 63 response (9 variables), (3) IFN-I: day 3 and day 59 (2 variables) and (4) Prime + Boost (13 variables). Performance was quantified using the area under the receiver operator curve in 5-fold cross-validation (CV-AUC). Analysis was focused on prediction of the CD4+ T-cell response at day 224 measured by PBMC-ICS and WB-ICS. Consistent with the correlation analysis, prediction of high responders could be predicted by the IFN-I responses 3 days after the prime and boost vaccinations (**Figure 9**). For WB-ICS the IFN-I variables had a CV-AUC = 0.91 (95% CI 0.78 – 1.00) and the addition of other variables did not improve prediction. The IFN-I variable set was also predictive of the PBMC-ICS CD4+ T-cell response with CV-AUC = 0.71 (95% CI 0.45 – 0.96).

## Discussion

ID93+GLA-SE elicits a multitude of immune responses that can be detected as differentially regulated genes in peripheral blood, 3 and 7 days after vaccination. Using a modular gene analysis approach we mapped modules of correlated genes to their associated cell types and functions. From the vaccine response signature it was apparent that a diverse population of cells was responding, with innate immune populations, such as eosinophils, neutrophils, and monocytes responding 3 days post-vaccination and a mix of innate and adaptive populations responding by day 7. The gene expression responses seemed to be attributable to both changes in the relative composition of immune cell types in the blood as well as changes in their gene expression; for example, while dendritic cells and memory B cells were increased 3 and 7 days after vaccination (respectively), several modules contained a relatively select set of genes with increased expression that could be more easily explained by a changed gene expression pattern. To our knowledge this is the first human transcriptomic analysis of the GLA-SE adjuvant and the ID93+GLA-SE vaccine.

Pre-clinical studies have previously demonstrated the efficacy of therapeutic immunization with ID93+GLA-SE to improve TB treatment outcomes (30, 31); many of the innate and adaptive signatures we observed in this study are consistent with the immune responses observed in model systems. A prior study of GLA-SE in mice at 6 hours, 1 day, 3 days and 7 days post-injection identified DEGs that were enriched for genes involved in innate antiviral responses and the type-I IFN pathway as early as 6 hours post-vaccination (32). Adaptive responses in the mouse related to T cells, cytokines, antigen presentation and cell cycle were evident from day 3 through day 7, earlier than we observed in humans.

It was notable that the responses of innate modules 3 days after the booster dose (day 59) were stronger than the responses 3 days after the prime (day 3); this was evidenced by the larger number of differentially expressed genes, and larger shifts in expression. A similar phenomenon was observed in studies of the adjuvant AS01_B_ and AS01_E_, which contain the TLR4-agonist MPL (33–35). In those studies, blood transcriptomic responses 1 day after vaccination were characterized by an increase in genes associated with dendritic cells (DCs), monocytes, neutrophils and IFN signaling; responses 1 day after the 2^nd^ dose were greater than responses 1 day after the 1^st^ dose. This could be an effect of trained immunity, which refers to long-term epigenetic reprogramming of innate cells that can alter responsiveness for months. Effects of trained immunity have been demonstrated with live-attenuated vaccines and hypothesized with TLR-stimulating adjuvants such as GLA-SE (36). Alternatively, the mechanism could involve CD4+ memory T cells that respond quickly after the boost or prime-induced antibody immune complexes that help to enhance the innate response (34). Notably, participants in this study had some level of pre-existing ID93-specific T-cell memory at baseline, whereas participants in the AS01 studies were naïve to the antigen (hepatitis B surface antigen, HBs).

Prior work has established that vaccine-induced immune responses are typically more robust among females; this has been shown for a variety of vaccine platforms and applies to both adaptive as well as innate responses (37). Among others, genes associated with viral sensing and the type-I IFN response are often enhanced in females, demonstrated recently in a study of replication defective herpes simplex virus 2 vaccine (38). In this study, the vaccine-induced increase in the IFN-I and EOS modules at day 3 was more robust among the female vaccine recipients. Female participants had higher ID93-specific IgG and CD4+ T cell responses after the first dose, consistent with the increased type-I IFN response and the correlations between the two. After subsequent doses sex was no longer associated with higher CD4+ T cell and IgG responses; it is possible that multiple vaccine doses reduced the effects, however a limitation of the study was the sex-imbalance in the three-dose vaccine group, among those that had samples available for transcriptional analysis. This reduced our ability to measure the effects of sex on late adaptive responses.

Our correlative analysis of the transcriptomic and adaptive responses revealed a moderate positive association between the day 3 IFN-I and EOS modules and the antigen-specific CD4+ T-cell response; remarkably the correlation was consistent across two samples/assays (PBMC or whole-blood ICS) and from 2 weeks to 7 months after the 1^st^ dose. The IFN-I response at day 59 also showed a similar trend suggesting that each participant’s ability to mount a type-I IFN response was generally a predictor of their CD4+ T cell response. The correlations of IFN-I with CD4+ T cell and IgG responses were weaker when we analyzed baseline-subtracted measures for these responses; it suggests that it’s not the pre-existing T cell response that is driving the IFN-I vaccine response, but all these results require validation in a larger cohort.

With our attempt to classify high versus low CD4+ T-cell responders we found that the innate IFN-I response could predict the Day 224 CD4+ T cell responders with an AUC of 0.91 (WB-ICS) and 0.71 (PBMC-ICS), and outperformed combinations of the other transcriptional response modules. The ability to predict a durable and robust T cell response from an innate gene signature is quite promising; such a signature could be used to accelerate the design or selection of adjuvants with improved durability. As this study enrolled a relatively small, geographically specific cohort, future studies will be needed to validate the predictive performance and understand the immune mechanisms. The prior studies of AS01 with hepatitis B virus surface antigen (HBs antigen) similarly showed a positive correlation between the innate response and the HBs-specific CD4+ T cell response (39). A study of RTS,S/AS01 in malaria-naïve adults also reported similar transcriptomic responses with type-I IFN related genes increased at day 1 and plasmablast and cell-cycling genes increased at day 6, however correlations with the CD4+ T cell response were only observed within a group that received an initial dose of an Ad35-vectored immunogen (35).

In the AS01 studies, plasmablast-associated genes peaked at day 7 (or day 6 with RTS,S), yet both the HBs/AS01 and RTS,S/AS01 studies found a positive correlation between expression of plasmablast-associated genes at day 1 with the antibody response two weeks later. The BCELL module we identified was not upregulated at day 3, nor was it correlated with the absolute level of ID93-specific IgG (we did not measure gene expression at day 1). However, we did observe that the response of the BCELL module 7 days after the boost was positively associated with the relative increase in IgG following the boost. This positive association of a plasmablast gene signature with the magnitude of the antibody response has been seen with several other vaccines (40), including the influenza tetravalent inactivated vaccine (TIV) (41) and yellow fever YF-17D (42).

A unique feature of the TBVPX-203 study was that it provided a direct comparison of immune responses after a final boost dose at day 56, with or without an intervening dose at day 28. We found that the response of the MITOSIS module at day 59 was significantly diminished in the 3-dose compared to the 2-dose group. The module, which was enriched for cell cycling and mismatch repair genes, was similar to responses observed 7 days after vaccination with AS01 (34, 35) and a variety of other vaccine adjuvants and platforms (40), and seemed to be associated with expansion of the T and B lymphocytes. In this study the decrease in response with the 3-dose schedule is consistent with the small or negligible subsequent increase in antibody titers and frequency of CD4+ T cells. Trends for a similar pattern in the NEUTRO-I and IFN-I modules 3 days after the boost (day 59) suggest that the innate response was also diminished with the 3-dose schedule. These data further bolster the decision that was made to advance the 2-dose regimen for further clinical testing. The lack of samples after the intervening boost at day 28 and at day 7 post-prime were limitations of the study that prevented us from more fully characterizing the effect of the vaccine schedule on immunogenicity. Though further experiments would be needed to describe the mechanisms underlying the effects of dose interval on immunogenicity, our observation is consistent with a recent review of vaccine RCTs that compared dose schedules; the review showed that longer dose intervals have been associated with increased antibody responses in trials of mRNA COVID-19, AS04-adjuvanted HPV and inactivated poliovirus vaccines (43).

The IFN-I module in this study included several genes that also appear in gene signatures used to predict risk of TB disease progression (26, 28). As expected, the risk signature scores in these participants track closely with the IFN-I module, with a transient increase 3 days post-vaccination and subsequent resolution by day 7. A recent study of H56:IC31, a subunit vaccine with a TLR9-stimulating adjuvant, assessed immunogenicity of the vaccine administered as adjunctive immunotherapy during TB treatment (44). In that study the TB risk signature genes increased expression two months after treatment completion and the increase was greater among placebo recipients compared to H56:IC31; samples were not available to establish when the gene signature initially increased or whether or not it ever resolved. With ID93+GLA-SE vaccination within 28 days after treatment completion we see only transient vaccine-induced increases and no evidence of long-term changes in TB risk scores over 18 months, suggesting there may not have been a similar post-treatment increase, however we have no population-matched healthy controls with which we can directly evaluate absolute TB risk scores.

Our data provide a window into how the innate and adaptive responses are orchestrated after vaccination with ID93+GLA-SE in a population that was recently treated for TB disease. The transcriptional responses provided an opportunity to dissect the combined effects of host sex, vaccine schedule and adjuvant dose on immunogenicity. A limitation of this study and many vaccine studies is the lack of an adjuvant-only or empty-vector group that would allow for further dissection of the innate and adaptive responses; determining the role of recall responses was particularly challenging in this study since participants were not naïve to the *M.tb* antigens in the vaccine, even at baseline. Though we have dissected and described the immune responses to ID93+GLA-SE, we provide no new evidence about which aspects of the immune response may or may not be protective; an ongoing phase 2b study of therapeutic vaccination with ID93+GLA-SE for prevention of TB recurrence could be an opportunity to validate the response modules we observed and potentially evaluate them as correlates of protection (HVTN603/ACTG5397, NCT06205589). There may also be an opportunity to identify common correlates of protection with M72/AS01_E_, since the two evidently induce similar innate responses; identifying correlates of protection for a disease can accelerate clinical development through greater understanding of mechanism, and by establishing surrogate immunogenicity endpoints for smaller, faster trials (45). Beyond TB, the response signatures of ID93+GLA-SE and the effects of dose schedule may be informative for the continued development of GLA and GLA-SE as an adjuvant for other vaccines such as those for preventing HIV-1 (46), leishmaniasis (47), leprosy (48), schistosomiasis (49), and malaria (50).

## Data Availability

The datasets presented in this study can be found in online repositories. The transcriptomic dataset generated for this study can be found in the NIH SRA with Accession (PRJNA1101039). Analysis datasets have been made public at: https://figshare.com/s/5e70a07df9bd2c359520. All analysis code for this manuscript can be accessed at https://github.com/agartland/TBVPX203_RNA.
