# Supplementary material for: Co-regulation of innate and adaptive immune responses induced by ID93+GLA-SE vaccination in humans
Source: Front Immunol. 2024 Sep 24;15:1441944. doi: 10.3389/fimmu.2024.1441944 (PMC11458388; doi:10.3389/fimmu.2024.1441944)
Supplement: Supplementary file 1 [file DataSheet1.docx]

**Coregulation of innate and adaptive immune responses induced by ID93+GLA-SE vaccination in humans**

Fiore-Gartland et al. *Frontiers Immunology*, 2024

**Supplementary Datasets**

Supplementary_Dataset_S1_modeling.xlsx

Supplementary_Dataset_S2_adaptive_correlation_results.xlsx

**Supplementary Tables**

**Table S1. Cohort and sample availability**

| **Availability of samples** | **Study Day** | | | | | | |
| --- | --- | --- | --- | --- | --- | --- | --- |
| **Treatment Group** | **0*** | **3** | **56*** | **59** | **63** | **112** | **168** |
| 10 µg ID93 + 2 µg GLA-SE | 5 | 5 | 0 | 5 | 5 | 2 | 2 |
| 2 µg ID93 + 2 µg GLA-SE | 15 | 14 | 0 | 13 | 13 | 2 | 2 |
| 2 µg ID93 + 5 µg GLA-SE (2-dose) | 15 | 14 | 12 | 11 | 11 | 10 | 9 |
| 2 µg ID93 + 5 µg GLA-SE (3-dose) | 14 | 12 | 9 | 9 | 10 | 8 | 7 |
| Placebo | 12 | 12 | 4 | 12 | 12 | 4 | 4 |
| **TOTAL** | 61 | 57 | 25 | 50 | 51 | 26 | 24 |

*vaccine administration in at least one group

**Supplementary Figures**

| **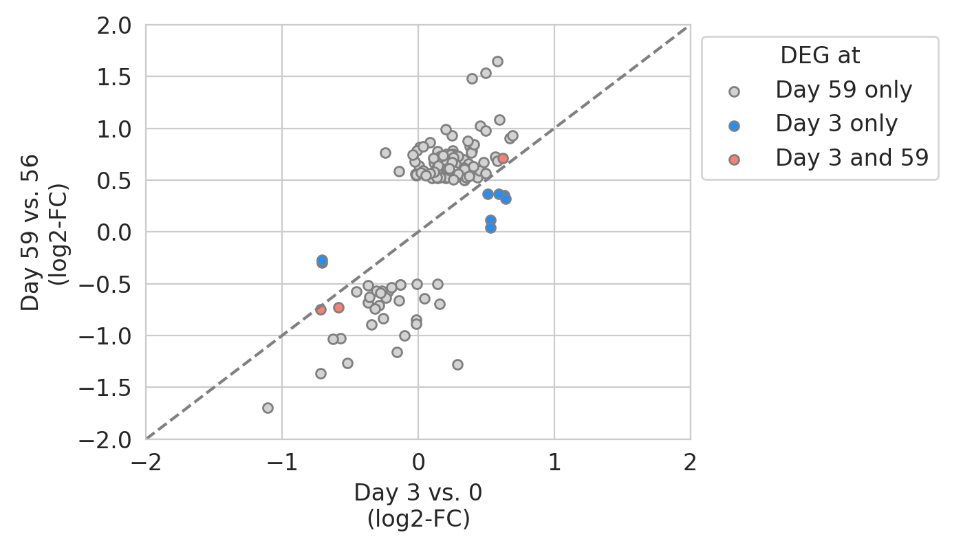** |
| --- |
| **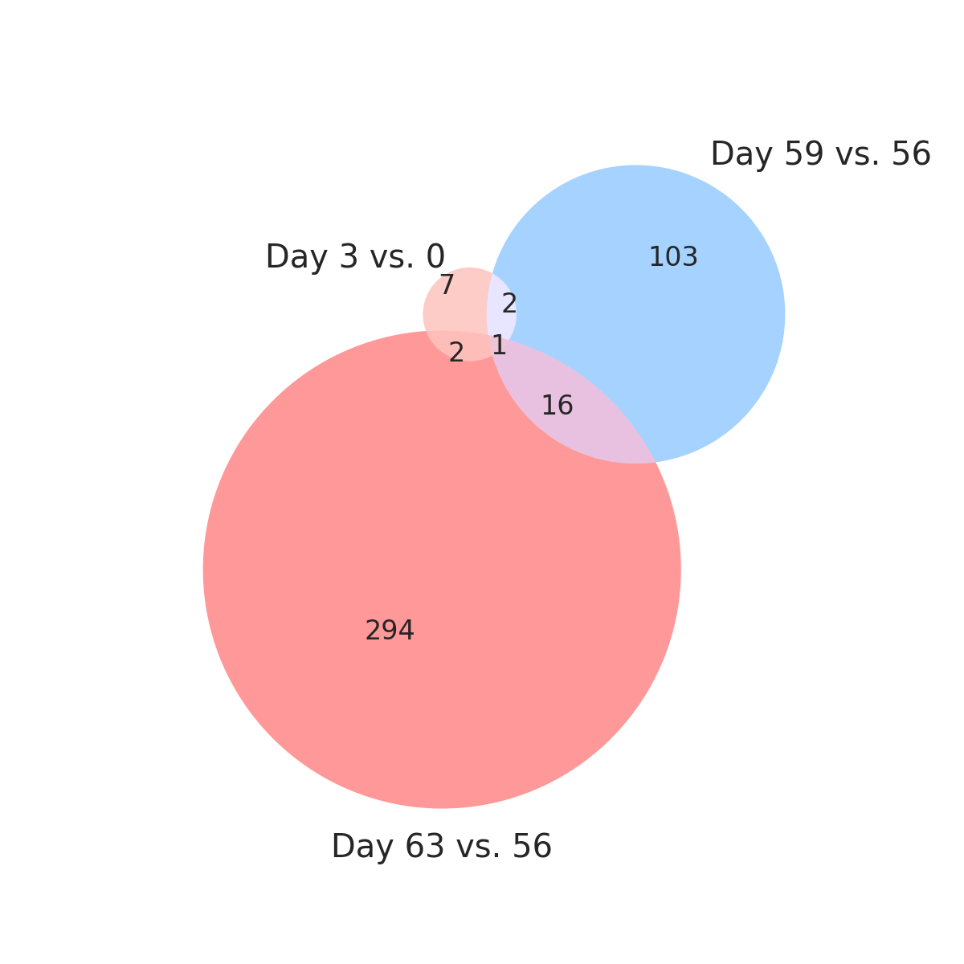** |

**Figure S1. Comparison of differential gene expression at multiple time points.** Models evaluating differential gene expression for day 3 vs. 0 and for day 59 vs. 56 were compared by plotting the log fold-change of each gene (TOP; limited to genes that were significantly differentially expressed for one of the two comparisons). Dashed line indicates equal fold-change for the two comparisons. Venn diagram shows the overlap of genes that were differentially expressed at multiple time points (BOTTOM).

| 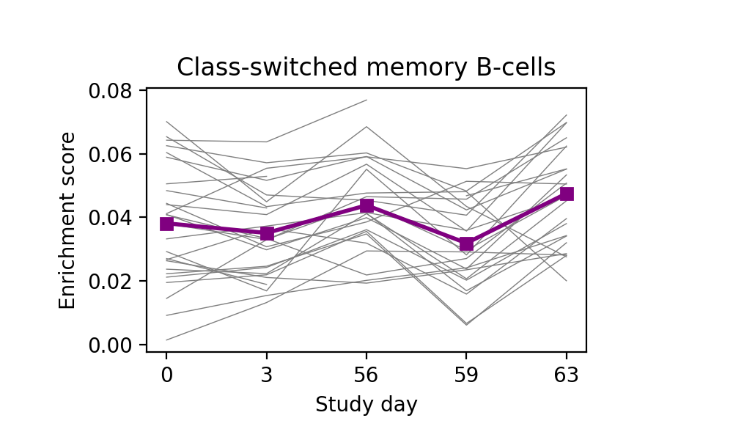 | 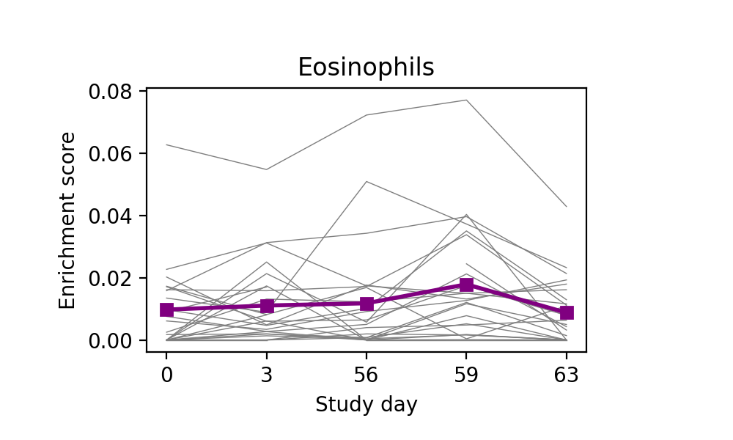 |
| --- | --- |
| 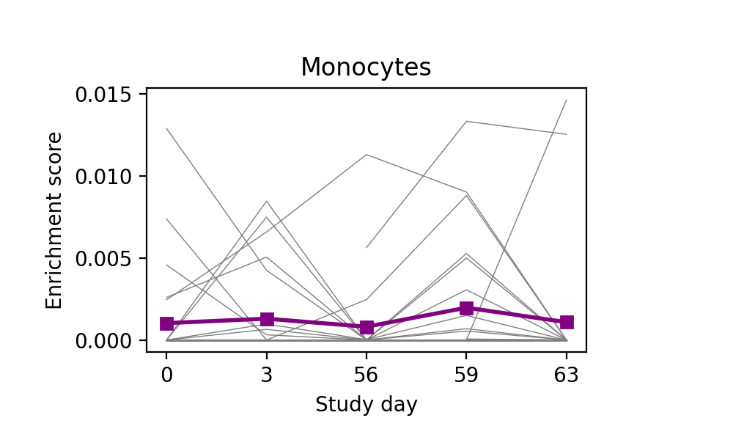 | 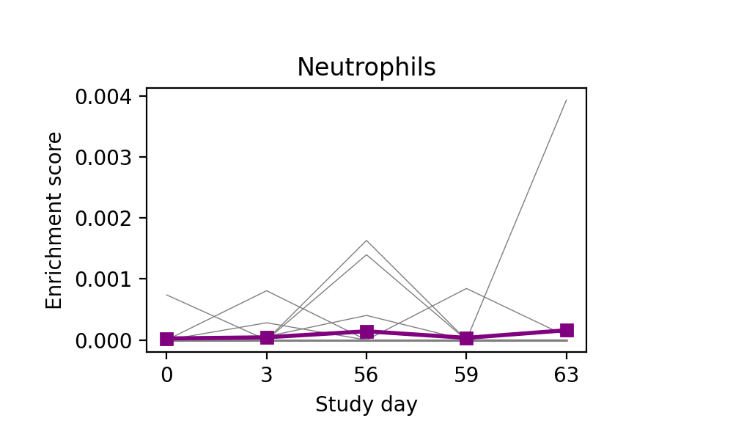 |
|  |  |
| 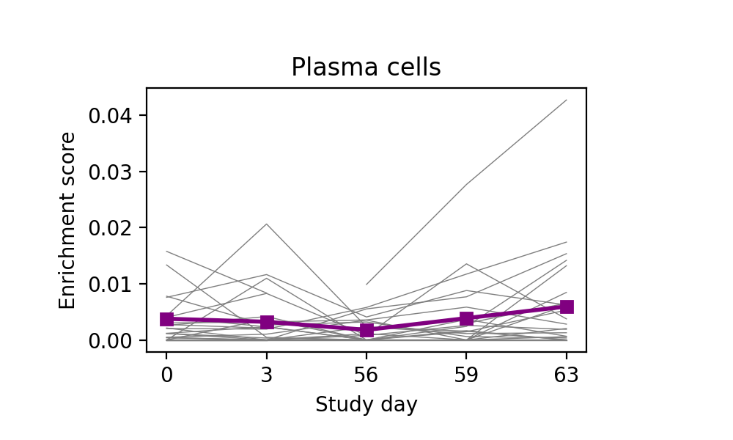 | 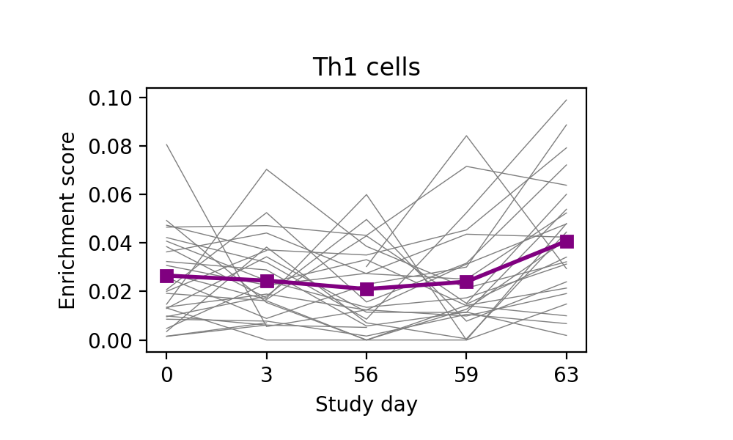 |
| 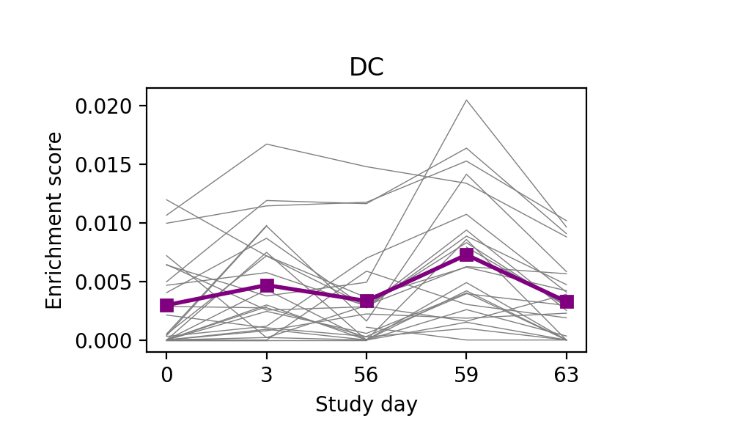 | **Figure S2. Deconvolution of relative proportion of immune cell subsets from bulk transcripts.** Estimation of the enrichment scores of immune cell subsets in blood was conducted using xCell(1) (see Methods). The score for each participant in either the 2-dose or 3-dose “2 + 5” regimen is represented as a thin line (gray). Thicker line (purple) is the group mean. Full xCell results and statistical testing are presented in Supplementary Dataset S1. |

| 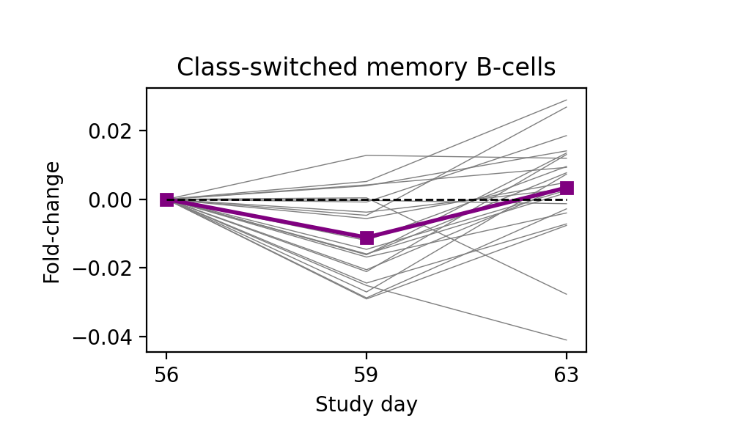 | 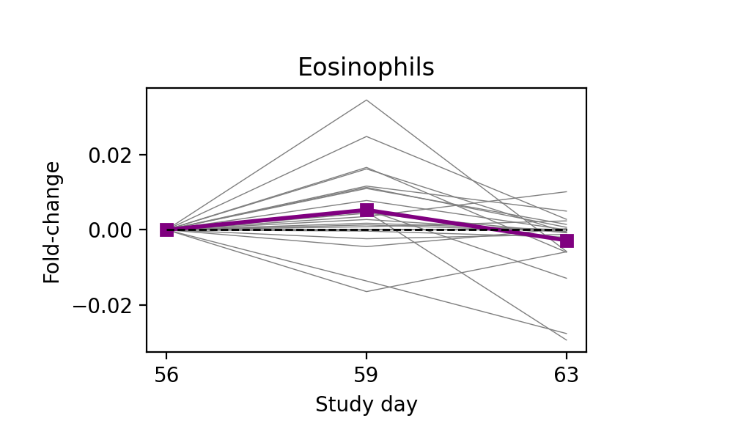 |
| --- | --- |
| **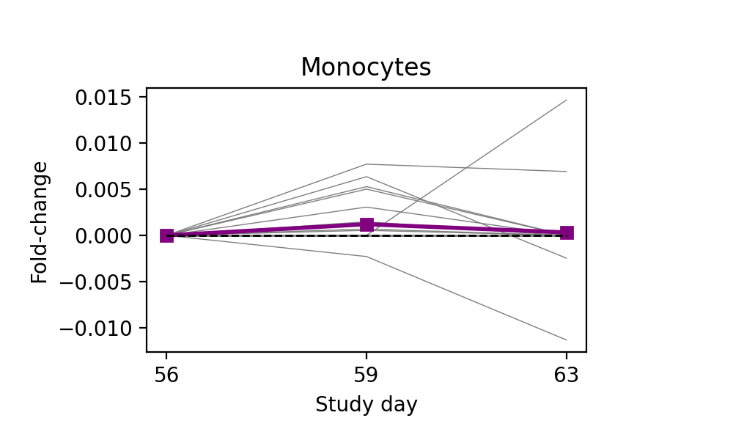** | **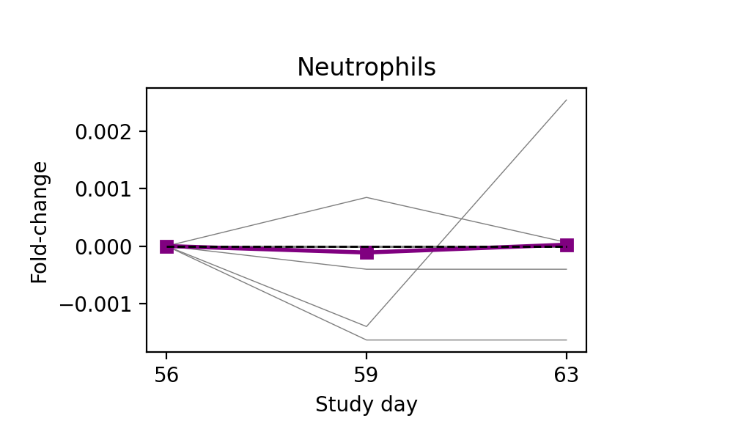** |
| **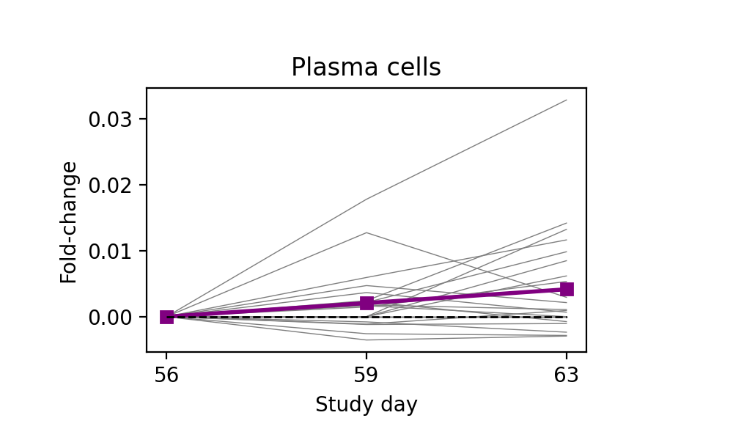** | **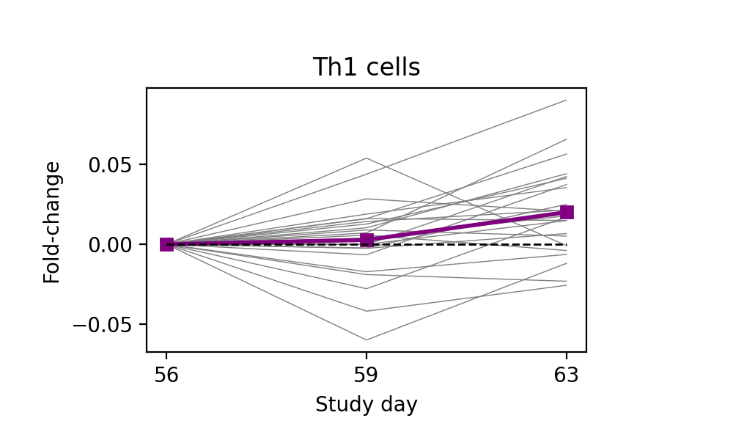** |
| **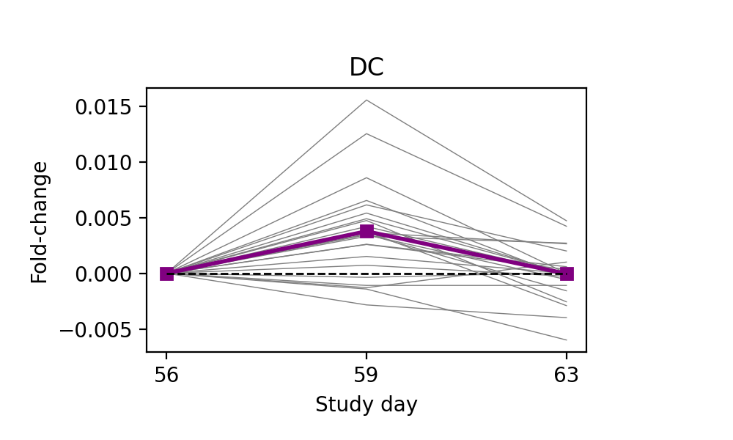** | **Figure S3. Deconvolution of relative proportion of immune cell subsets from bulk transcripts.** Estimation of the enrichment scores of immune cell subsets in blood was conducted using xCell(1) (see Methods) plotted as a fold-change relative to day 56. The score for each participant in either the 2-dose or 3-dose “2 + 5” regimen is represented as a thin line (gray). Thicker line (purple) is the group mean. Full xCell results and statistical testing are presented in Supplementary Dataset S1. |

| **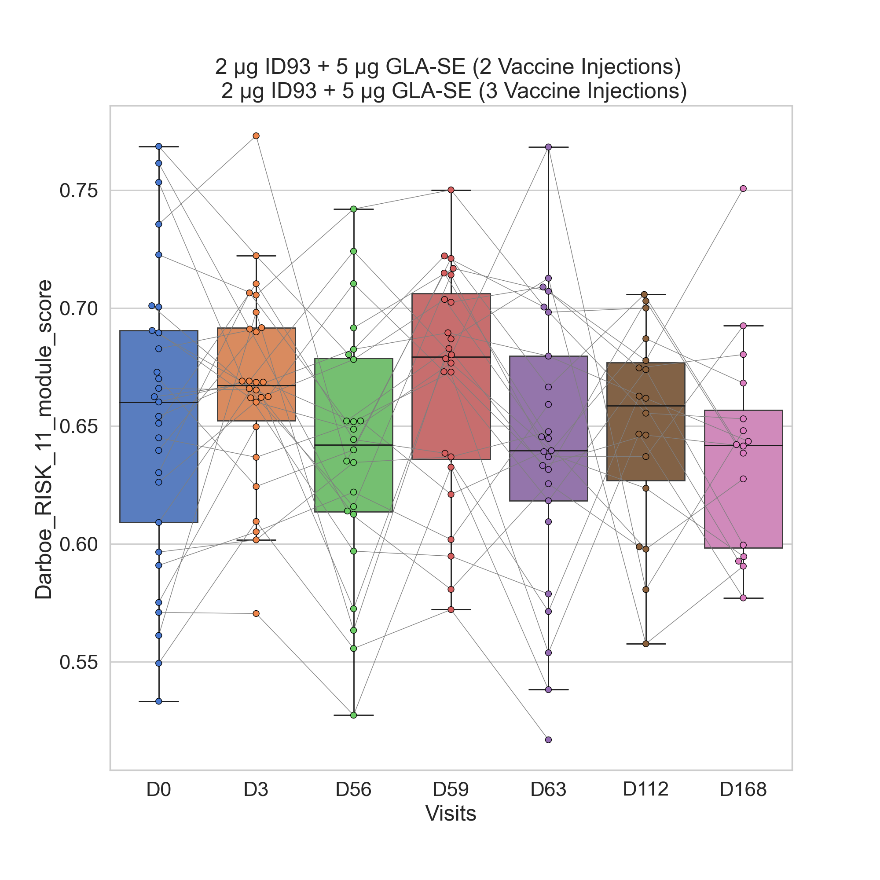** |
| --- |
| **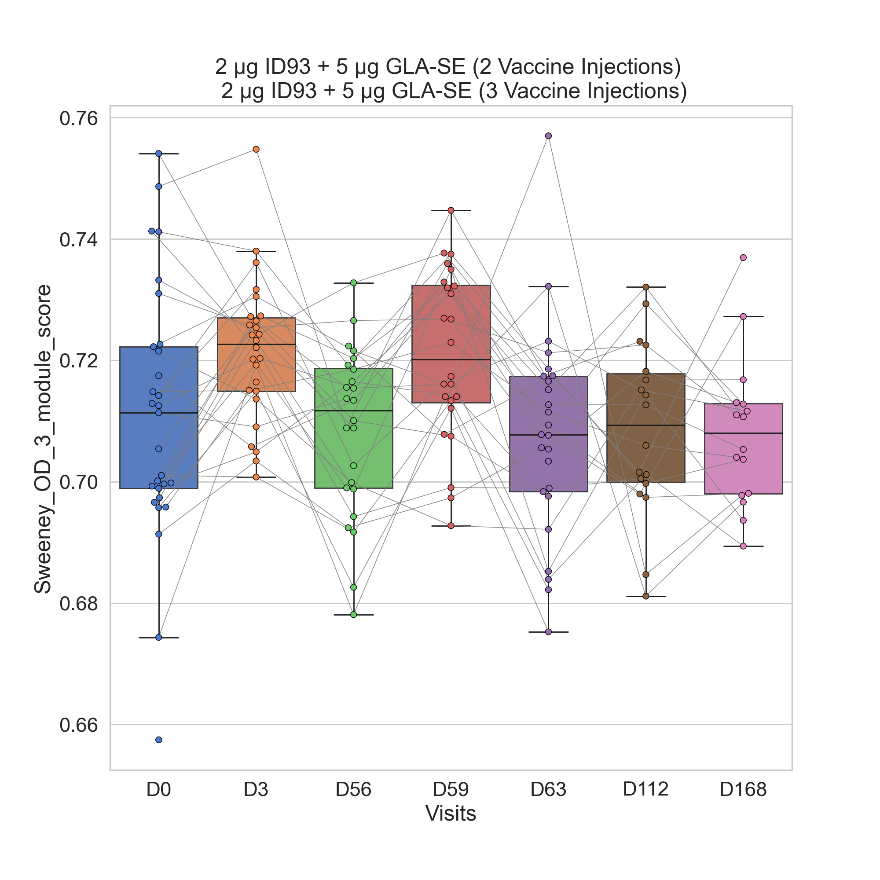** |

**Figure S4. Longitudinal expression of genes included in two TB disease risks scores**

TB disease risk scores Darboe11 (TOP PANEL) and Sweeney3 (BOTTOM PANEL) computed from normalized counts from the longitudinal RNA sequencing data (all ID93+GLA-SE recipients, any group). Each line connecting longitudinal data indicates one participant. Boxplots indicate median and interquartile range of the scores at each day. Whiskers extend to the maximum data value within 1.5 times the interquartile range. Scores computed using R package *TBSignatureProfiler* (3).

**
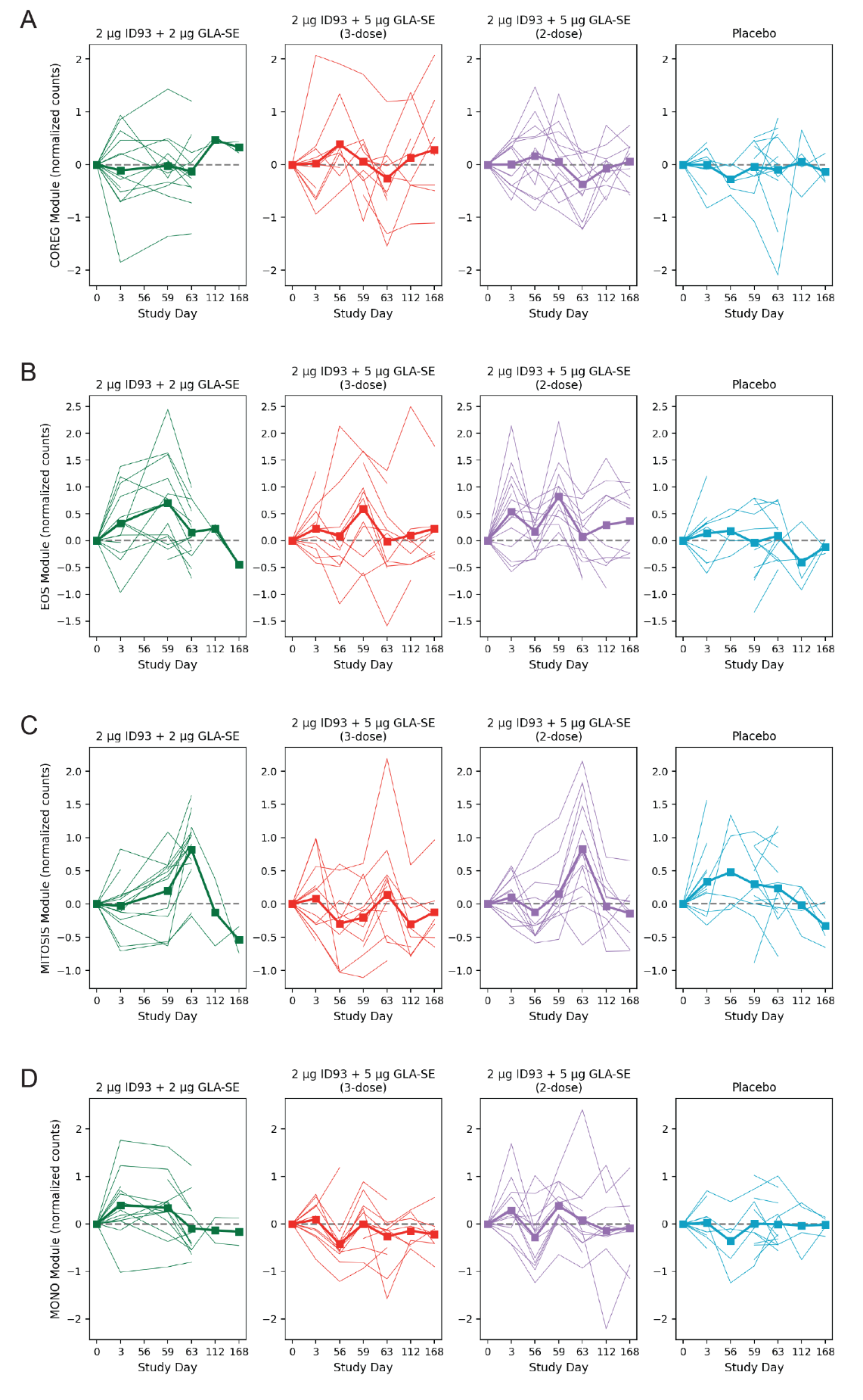
**

**
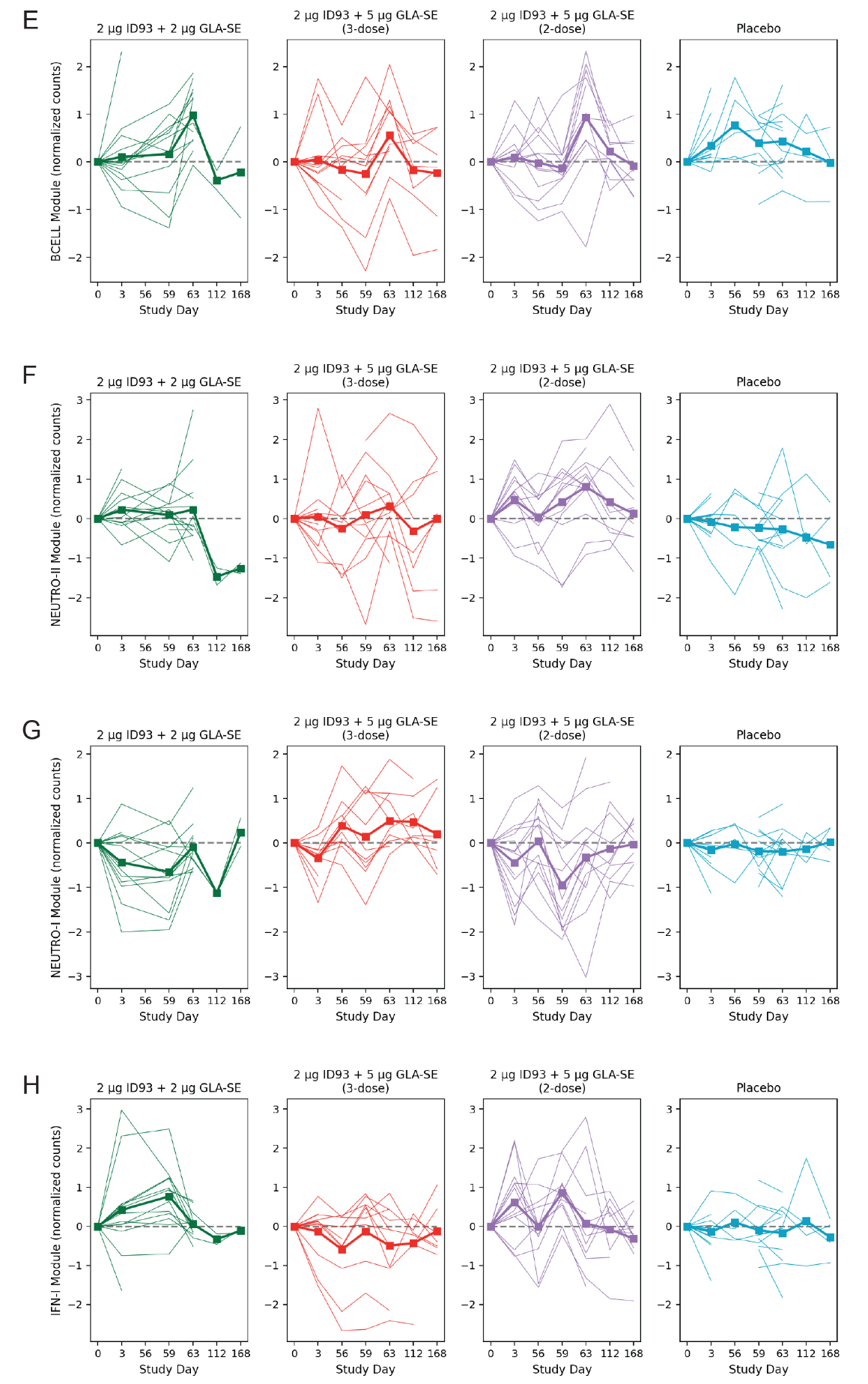
**

**Figure S5. Longitudinal module gene expression by treatment group**

Baseline-subtracted normalized expression level is plotted for each module, participant, and day. Module expression is computed as the log-normalized transcript counts with a mean taken across all genes contained in a module; each participant’s module expression at day 0 is subtracted from the time series. Module expression values are plotted longitudinally with each participant represented by a single line and the group mean represented by a thick line and square symbols (treatment group indicated at the top of each panel). Each of the eight gene modules are plotted in panel rows A – H. Dashed line on each plot indicates zero change from day 0 (i.e., pre-vaccine) expression. Broken lines represent missing data that result from missing samples.

**
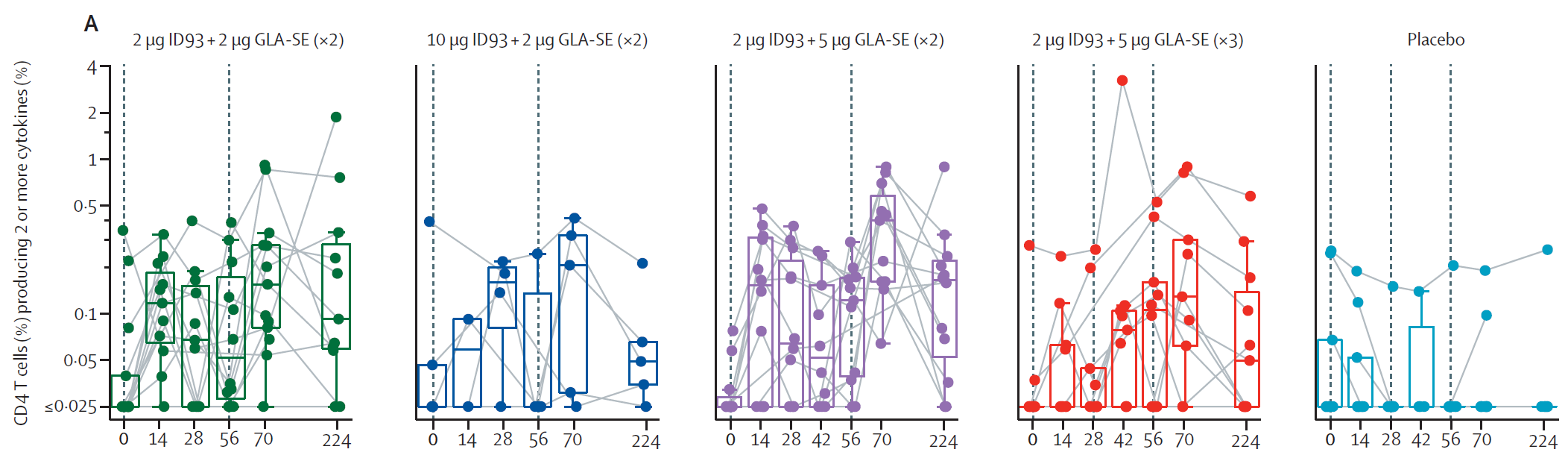
**

**Figure S6. ID93-specific CD4+ T cell responses measured by PBMC-ICS**

Reproduction of figure from Day T, Penn-Nicholson A, et al. (2) showing the percent of CD4+ T cells expressing ≥2 of IL2, IFNγ, TNFα, or CD154. Data are plotted by treatment group in the TBVPX-203 trial and censored at 0.025%.

**
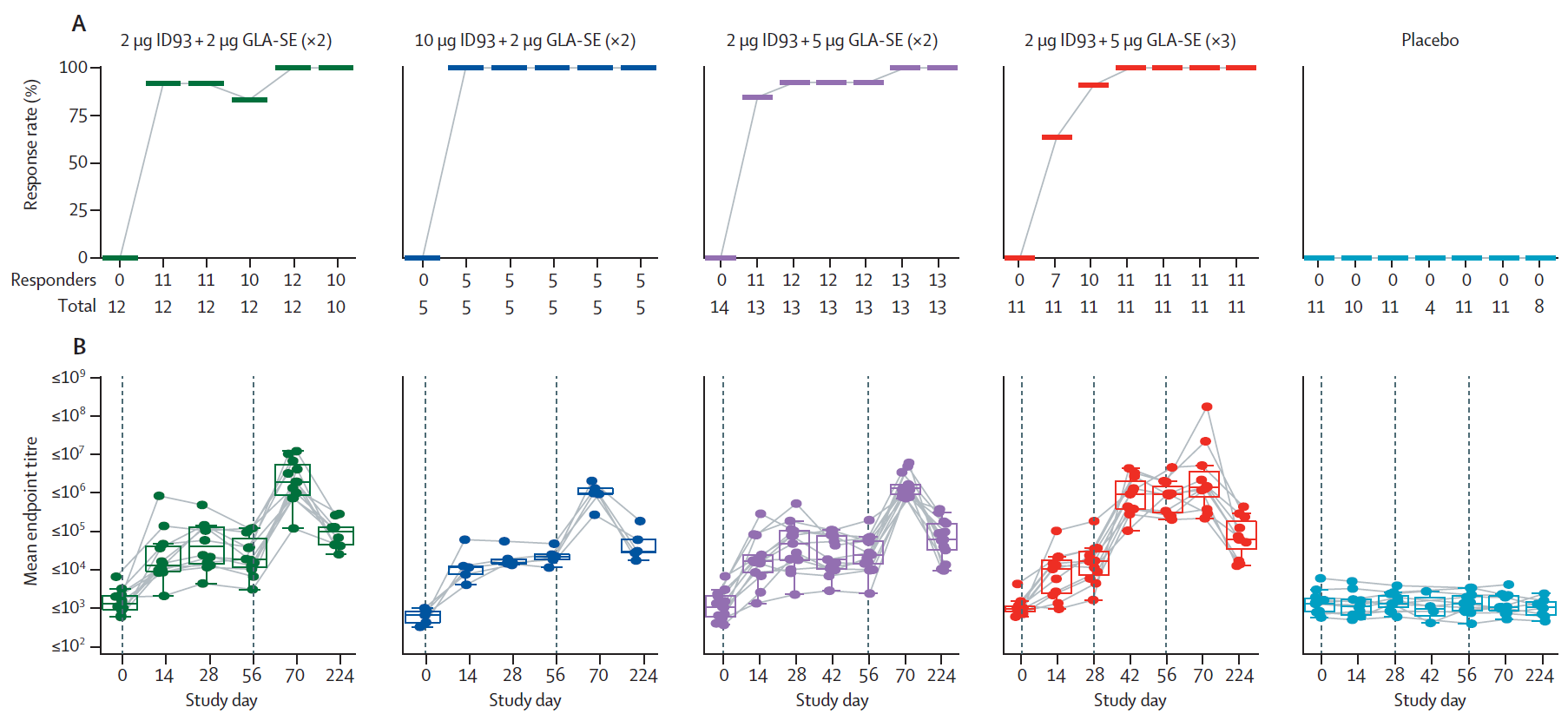
**

**Figure S7. ID93-specific IgG measured by ELISA**

Reproduction of figure from Day T, Penn-Nicholson A, et al. (2) showing the geometric mean endpoint titers (MEPT) for total anti-ID93 IgG, by treatment group in the TBVPX-203 trial.

| **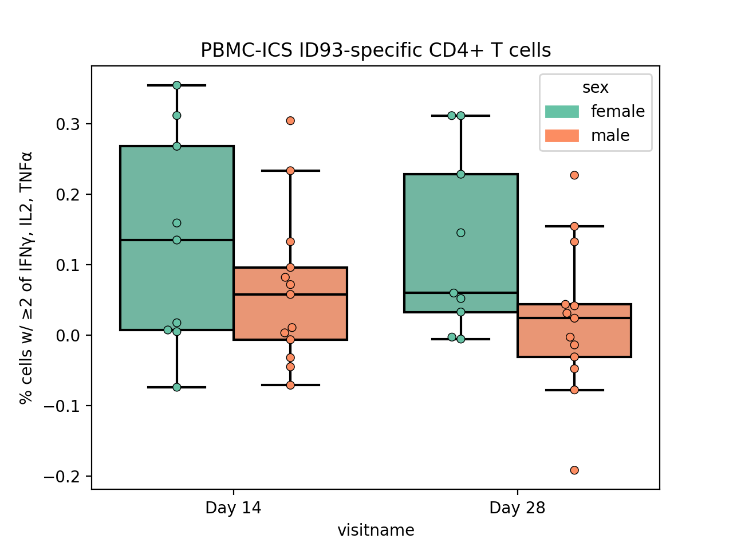** |
| --- |
| **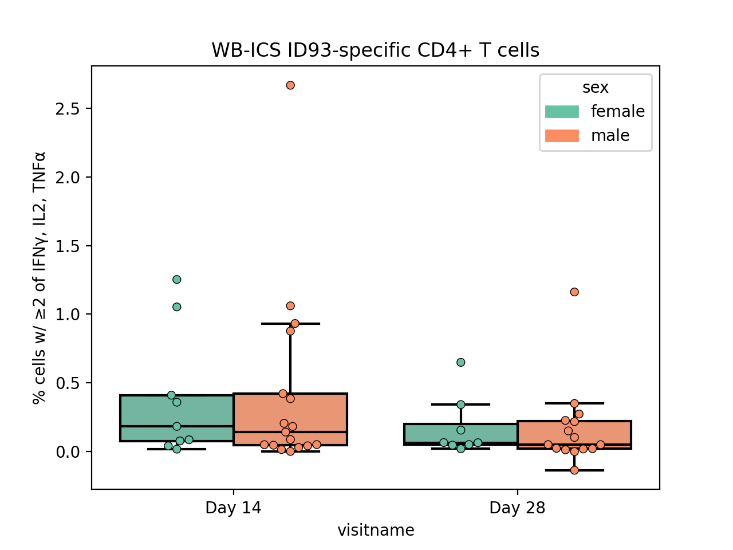** |
| **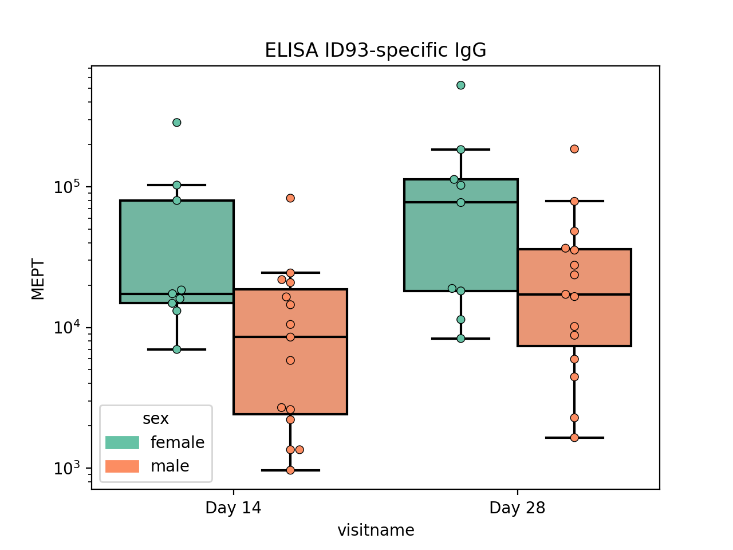** |

**Figure S8. Association of sex with adaptive vaccine responses**

Boxplots showing the ID93-specific CD4+ T cell responses and IgG mean endpoint titers (MEPT) among participants in the 2-dose and 3-dose treatment groups, split by sex. Responses were significantly higher among females at day 14 (2-weeks post-first dose) for the PBMC-ICS CD4+ T cell response (p = 0.032) and the ELISA IgG MEPT (p = 0.039).

| **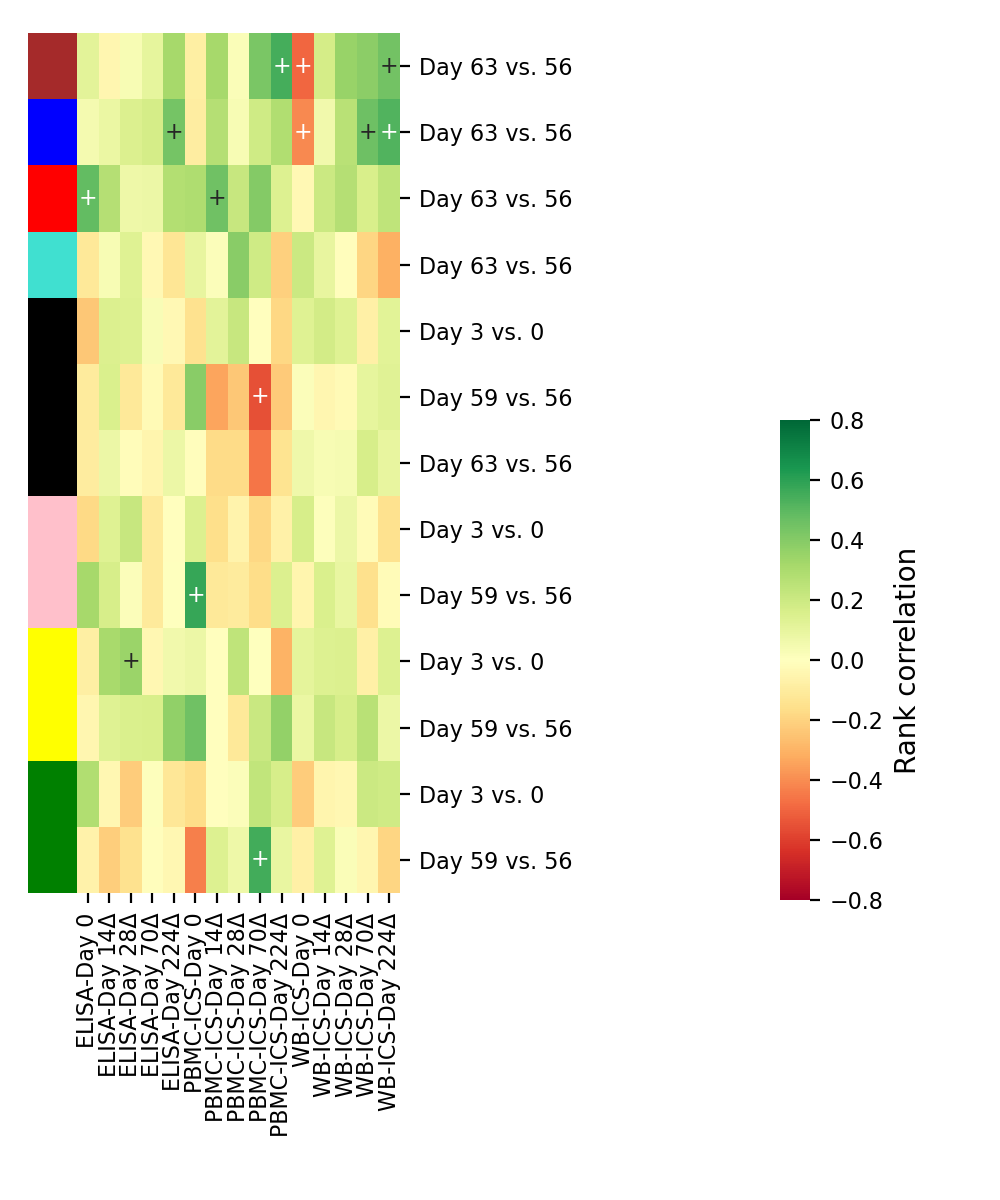** | **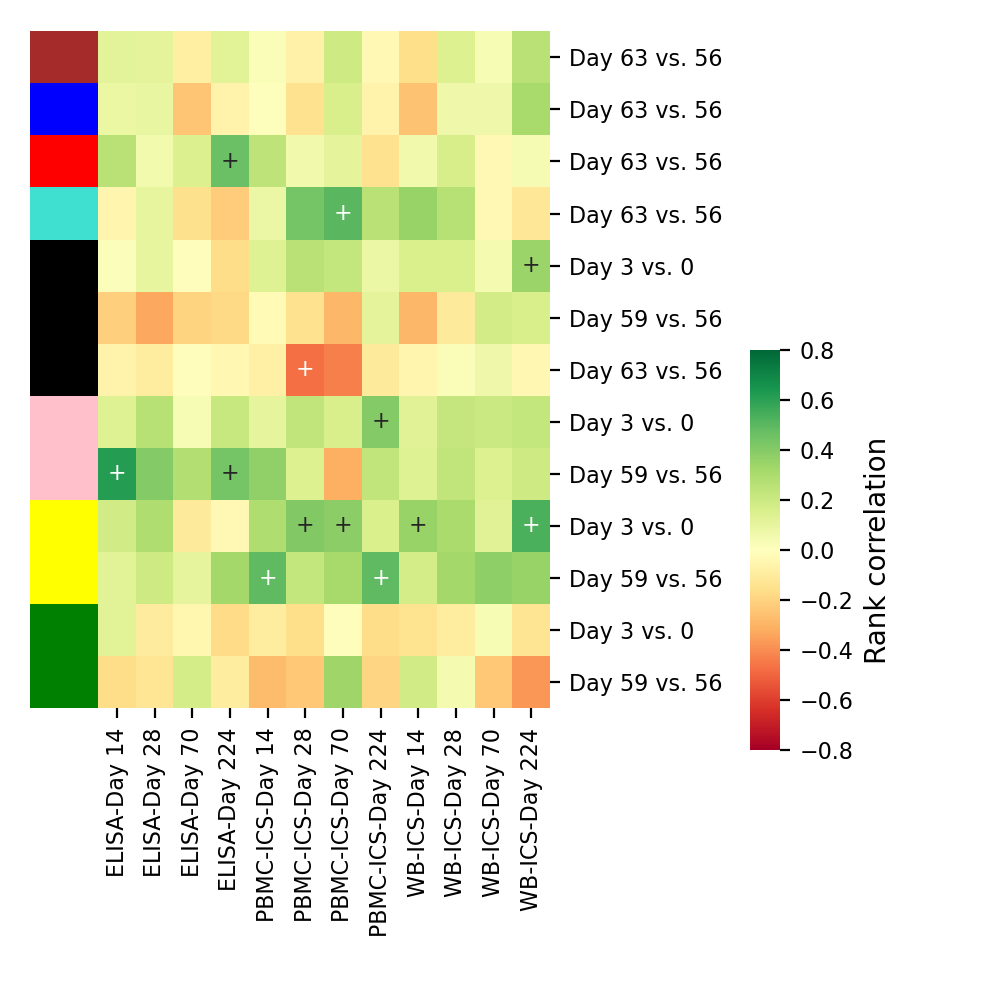** |
| --- | --- |

**Figure S9. Correlation between transcriptional and adaptive vaccine responses**

Rank correlation was estimated between pairs of adaptive immune responses (PBMC-ICS, WB-ICS and ELISA) and gene expression changes for the 13 module comparisons that showed a significant change. Plus symbol indicates a p-value < 0.05. Color bar along y-axis indicates the gene module, with the specific comparison on the right side. (LEFT PANEL) Adaptive immune responses were transformed by subtracting the response at the day of the last injection (day 0 or 56). Correlations reflect an association between a change in the gene module with a relative change in the adaptive response at the specified day. Absolute values at Day 0 were also included in this analysis. (RIGHT PANEL) Correlations computed as in Figure 7C with adjustment for participant sex. Correlation is computed between the absolute immune response at the specified visit (x-axis) with the change in gene expression of the module indicated on the y-axis.

**References**

1. Aran D, Hu Z, Butte AJ. xCell: Digitally portraying the tissue cellular heterogeneity landscape. *Genome Biol* (2017) 18:1–14. doi: 10.1186/s13059-017-1349-1

2. Day TA, Penn-Nicholson A, Luabeya AKK, Fiore-Gartland A, Du Plessis N, Loxton AG, Vergara J, Rolf TA, Reid TD, Toefy A, et al. Safety and immunogenicity of the adjunct therapeutic vaccine ID93 + GLA-SE in adults who have completed treatment for tuberculosis: a randomised, double-blind, placebo-controlled, phase 2a trial. *Lancet Respir Med* (2020) 2600:1–14. doi: 10.1016/S2213-2600(20)30319-2

3. Johnson WE, Odom A, Cintron C, Muthaiah M, Knudsen S, Joseph N, Babu S, Lakshminarayanan S, Jenkins DF, Zhao Y, et al. Comparing tuberculosis gene signatures in malnourished individuals using the TBSignatureProfiler. *BMC Infect Dis* (2021) 21: doi: 10.1186/S12879-020-05598-Z
